# Design of new molecules against cervical cancer using DFT, theoretical spectroscopy, 2D/3D-QSAR, molecular docking, pharmacophore and ADMET investigations

**DOI:** 10.1016/j.heliyon.2024.e24551

**Published:** 2024-01-24

**Authors:** Said El Rhabori, Abdellah El Aissouq, Ossama Daoui, Souad Elkhattabi, Samir Chtita, Fouad Khalil

**Affiliations:** a Laboratory of Processes, Materials and Environment (LPME), Sidi Mohamed Ben Abdellah University, Faculty of Science and Technology - Fez, Morocco; b Laboratory of Engineering, Systems and Applications, National School of Applied Sciences, Sidi Mohamed Ben Abdellah-Fez University, Fez, Morocco; c Laboratory of Analytical and Molecular Chemistry, Faculty of Sciences Ben M'Sik, Hassan II University of Casablanca, Morocco

**Keywords:** Cervical cancer, QSAR, DFT, Molecular docking, Pharmacophore, ADMET

## Abstract

Cervical cancer is a major health problem of women. Hormone therapy, via aromatase inhibition, has been proposed as a promising way of blocking estrogen production as well as treating the progression of estrogen-dependent cancer. To overcome the challenging complexities of costly drug design, in-silico strategy, integrating Structure-Based Drug Design (SBDD) and Ligand-Based Drug Design (LBDD), was applied to large representative databases of 39 quinazoline and thioquinazolinone compound derivatives. Quantum chemical and physicochemical descriptors have been investigated using density functional theory (DFT) and MM2 force fields, respectively, to develop 2D-QSAR models, while CoMSIA and CoMFA descriptors were used to build 3D-QSAR models. The robustness and predictive power of the reliable models were verified, via several validation methods, leading to the design of 6 new drug-candidates. Afterwards, 2 ligands were carefully selected using virtual screening methods, taking into account the applicability domain, synthetic accessibility, and Lipinski's criteria. Molecular docking and pharmacophore modelling studies were performed to examine potential interactions with aromatase (PDB ID: 3EQM). Finally, the ADMET properties were investigated in order to select potential drug-candidates against cervical cancer for experimental in vitro and in vivo testing.

## 1 Introduction

Cancer is one of the leading causes of death, making it a critical public health concern [1]. The situation was already complex, but it has been complicated by the obstacles to cancer diagnosis and treatment imposed by the COVID-19. Although cervical cancer is one of the most preventable types of cancer, it remains the second most common cause of cancer deaths in women under 50 [2]. This disease is a malignant tumor that develops in or on the lower part of the uterus and is divided into two basic categories depending on the type of cervical cells affected: cervical squamous cell carcinoma which affects the squamous cells of the cervix, and cervical adenocarcinoma which targets the mucus-producing cells of the cervix [3]. Most cases of cervical cancer follow infection with high-risk types of the genital human papilloma virus (HPV) [4,5]. The treatment of cancers associated with HPV, including surgical procedures, chemotherapy, and radiation therapy, may precipitate an array of undesirable side effects [6,7]. In addition to HPV, which is the predominant factor in the development of cervical cancer, hormonal elements have been identified as contributing factors. Indeed, estrogen is the main female reproductive hormone responsible for a variety of biophysiological processes in women whose cervix is highly sensitive to this hormone [[8], [9], [10]]. In addition, estrogen receptors in cervical tissue enable the cervix to respond to estrogen influencing cell growth [[11], [12], [13]]. Consequently, it has been found that estrogens and HPV interact [14]. In fact, the activation of estrogen through the transcriptional functioning of estrogen receptors alpha (Erα) may be enhanced or diminished by HPV gene products [15]. As a result, several discoveries have highlighted the role of estrogen in cervical cancer, leading to the discovery of anti-estrogen drugs by targeting specific enzymes [16]. Aromatase inhibitors are a promising alternative in such conditions because they reduce high estrogen levels [17,18]. In fact, this enzyme is found mainly in the ovaries, where it converts androgens into estrogens. In addition, it is an important enzyme in cervical cancer but not in the normal cervix [19,20]. Even though cancer cell resistance is an issue, multiple efforts are being made to produce efficient cervical cancer inhibitors by investigating diverse molecular structures as ligands of aromatase. Due to their wide availability and minimal side effects, quinazoline and thioquinazolinone derivatives are gaining in importance as anti-cancer molecules [21,22]. In addition, research has shown that these heterocyclic compounds have medicinal chemistry properties beyond their anticancer effects including analgesic, anti-hypertensive, anti-inflammatory, anticonvulsant, anti-diabetic, antibacterial and dihydrofolate reductase inhibitory effects [[23], [24], [25]].

The most costly aspects of pharmaceutical research and development include the discovery of new drug-candidates, their pre-clinical and clinical progression, and the investigations following authorization. In recent years, the integration of bioinformatics, molecular modelling and machine learning into the drug development process has achieved significant results in terms of accelerating the process and reducing the costs associated with the development of new drug-candidates [26]. Among these computational techniques, the modelling of quantitative structure-activity relationships in two and three dimensions (2D-QSAR and 3D-QSAR) is one of the most widely used methods in the field of medicinal chemistry. In addition, various molecular modelling techniques including molecular docking and pharmacophore have also made significant contributions in this field, in parallel with in silico investigations of pharmacokinetic properties [27]. In this research contribution, we have applied computer-aided drug design (CADD) methodologies, including structure-based drug design (SBDD) and ligand-based drug design (LBDD), to study a set of 39 quinazoline and thioquinazolinone derivatives that show a potential effect on the treatment of cervical cancer [20,21]. To achieve this objective, the supervised machine learning approach involved the use of multiple linear regression (MLR) with pertinent descriptors as well as partial least squares regression (PLS) on the basis of comparative molecular field analysis (CoMFA) and comparative molecular similarity index analysis (CoMSIA) to build robust 2D/3D QSAR models. Next, the reliable models were used to assess the suitability of the investigated and designed molecules through multivariate statistical analysis and to predict improved anti-cervical cancer activities. Subsequently, we investigated the potential aromatase binding site (PDB ID: 3EQM) and obtained information on the most active and least active molecules, as well as newly designed inhibitors, using a rigorously validated molecular docking technique [28]. In addition, the pharmacophore modeling was used to validate the type of interactions identified by molecular docking linked to the selected descriptors and CoMSIA fields from the 2D/3D QSAR analysis. Finally, the selected compounds were examined for their similarity to existing drugs and their ADMET properties (absorption, distribution, metabolism, excretion and toxicity) were investigated in silico for subsequent experimental evaluation in vitro and in vivo.

## 2 Material and methods

### 2.1 Studied molecules

In order to perform molecular modeling, the experimental anti-cervical cancer activities of thirty-nine quinazoline (Fig. S1) and thioquinazolinone (Fig. S_2_) derivatives that were collected from the literature and employed as independent variables (Table 1) [21,22].

**Table 1:** Structures and anti-cervical cancer activities of 39 quinazoline and thioquinazolinone derivatives (pIC_50_ = -log IC_50_).

N°	pIC_50_	Structure	N°	pIC_50_	Structure	N°	pIC_50_	Structure
**1**	**4.739**		**14**	**4.261**		**27**	**4.175**	
**2**	**4.648**		**15**	**4.190**		**28**	**5.003**	
**3**	**4.427**		**16**	**4.583**		**29**	**4.242**	
**4**	**4.874**		**17**	**4.687**		**30**	**5.122**	
**5**	**4.920**		**18**	**4.128**		**31**	**5.040**	
**6**	**4.462**		**19**	**4.143**		**32**	**4.336**	
**7**	**4.271**		**20**	**4.309**		**33**	**5.071**	
**8**	**4.114**		**21**	**4.038**		**34**	**5.170**	
**9**	**4.778**		**22**	**4.153**		**35**	**4.715**	
**10**	**4.299**		**23**	**4.069**		**36**	**4.855**	
**11**	**4.477**		**24**	**4.840**		**37**	**4.550**	
**12**	**4.099**		**25**	**4.374**		**38**	**4.138**	
**13**	**4.543**		**26**	**4.971**		**39**	**4.252**	

### 2.2 QSAR models

#### 2.2.1 2D-QSAR and data splits

Following energy optimization of each molecule via MM2 force field [29], the programs Chem3D V16 [30], ChemSketch 12 [31] and Marvin Sketch [32] were utilized in order to determine topological, physicochemical, and geometric descriptors. Furthermore, we implemented the program Gaussian 09 [33] utilizing the DFT (Density-functional theory) method with the B3LYP functional and the 6-311G basis to obtain quantum chemical descriptors (Table 2, Table S1 and Fig. S_3)_ [34].

**Table 2:** Software and calculated descriptors.

Software	Descriptors calculated
**Chemoffice3D**	Number of Hydrogen Bond Acceptors (**NHA**); Number of Hydrogen Bonds Donors (**NHD**); Partition coefficient for octanol and water (**LogP**); Number of bond rotations (**NRB**); Energy of Stretch-Bend (**SB**); Torsion energy (**T**).
**Gaussian 09**	Dipolar moment (**DM**); Highest occupied molecule orbital energies (**E**_**HOMO**_ and **E**_**HOMO-1**_); Lowest unoccupied molecular orbital energies (E_**LUMO**_ and E_**LUMO+1**_); Index of electrophilicity (**ω**); Bond distance between C and S (**Bond C–S**); Carbon's charge on the sulfur atom (**Q**); Charge of C_1_ (**Q**_**1**_) and charge of C_2_ (**Q**_**2**_) of benzene ring (fig. S_**3**_)

Principal component analysis (PCA) [35] was used to extract as much information as possible from the database and select chemical descriptors that might be used to develop QSAR models (Table S_1_). Next, the database was randomly divided into training and test sets with 80 % and 20 % of data set, respectively [36].

#### 2.2.2 Statistical analysis and selection of 2D-QSAR models

Multiple linear regression (MLR) technique [37,38] was applied to develop 2D-QSAR models using XLSTAT V. 2014 [39]. The coefficient of determination $R^{2}$ (Eq. (1)), the adjusted coefficient determination $R_{adj}^{2}$ (Eq. (2)), the mean squared error MSE (Eq. (3)), the Fisher's statistical parameter (F-value) and the level of significance (p-value) are the primary parameters used in statistical analysis of QSAR models [[40], [41], [42]]:

(Eq. 1) $$R^{2}=1-\frac{\sum_{i=1}^{n}{\left({Y_{i}}_{obs}-{Y_{i}}_{cal}\right)}^{2}}{\sum_{i=1}^{n}{\left({Y_{i}}_{obs}-{\bar{Y}}_{cal}\right)}^{2}}$$

(Eq. 2) $$R_{adj}^{2}=\frac{\left(n-1\right)\times R^{2}-P}{n-1-p}$$

(Eq. 3) $$MSE=\frac{1}{n}\sum_{i=1}^{n}{\left({Y_{i}}_{obs}-Y_{cal}\right)}^{2}$$

Where $Y_{iobs}$ represents the value of the observed response i, $Y_{cal}$ is the value of the predicted response i, ${\bar{Y}}_{cal}$ represents the average value of predicted responses, P is the number of explicative variables in the model and n represents the number of molecules in the training set.

#### 2.2.3 Molecular alignment and CoMFA/CoMSIA models generation

The alignment of molecules is a crucial step in the development of the CoMFA and CoMSIA models [43]. For this, the process was initiated by sketching molecular structures using the SYBYL-X.2.1 platform and followed by their optimization using the Tripos force field and Gasteiger-Hückel atomic partial charges [44]. Using Powell's gradient approach, a considerable number of 100,000 iterations were required to obtain a stable conformation [45].

The CoMFA model was developed using steric (Str) and electrostatic (Elec) fields, generated by the sp^3^ hybridization of a carbon atom with a Van Der Waals radius of 1.52 and a net charge of 1.0 [46]. In comparison, the CoMSIA model uses a probe atom to generate more precise physicochemical descriptors, including hydrophobic (Hyd), hydrogen bond donor (HBD) and acceptor (HBA) fields, as well as steric and electrostatic fields. To achieve this, initial attenuation factors were set at 0.3 kcal/mol and column filtering was set at 2.0 kcal/mol [47]. Based on the partial least squares (PLS) method and taking into account the results relating to the number of principal components (N), several models may be developed to explore the correlation between CoMFA and CoMSIA descriptors and anti-cervical cancer activity levels.

#### 2.2.4 Models’ validation

To evaluate the global significance of the models, several statistical parameters such as the coefficient of determination ($R^{2}$), the Fischer test (F-value) and the standard error of estimation (SEE) were applied as well as the highest coefficient of determination in cross-validation ($R_{cv}^{2}$) using the leave-one-out cross-validation (LOOCV) technique [48]. According to Eq. (4), the value more than 0.5 was chosen as the criteria for a favorable $R_{cv}^{2}$ value [49].

(Eq. 4) $$R_{cv}^{2}=1-\frac{\sum_{j=1}^{N}{\left(Y_{jobs}\left(train\right)-Y_{jcal}\left(train\right)\right)}^{2}}{\sum_{j=1}^{N}{\left(Y_{jobs}\left(train\right)-{\bar{Y}}_{cal}\left(train\right)\right)}^{2}}$$

Where: $Y_{jobs}^{2}$ (train) is the observed response's value, $Y_{jcal}$ (train) is the value predicted by Loo-cv and ${\bar{Y}}_{cal}$ (train) is the mean of predicted response values.

To evaluate the predictive performance of 2D/3D-QSAR models, the anti-cervical cancer activities of the molecules included in the test set have been estimated. For this, the external coefficient of determination $R_{test}^{2}$ was used as a measure to evaluate the reliability of the generated models [50].

To ensure the reliability of the QSAR model, Y-Randomization test has been performed. As part of this procedure, Y (the response parameters) are chosen at random with respect to the original X (matrix of descriptors) [51]. The randomized model's correlation coefficient ($R_{yrand}^{2}$) must be smaller than the non-randomized model's $R^{2}$ and an additional parameter ${\;CR}_{p}^{2}$ (Eq. (5)) must be more than 0.5:

(Eq. 5) $$cR_{p}^{2}=R^{2}\times \sqrt{(R^{2}-}R_{yrand}^{2})$$

An approach based on values of $Q_{yrand}^{2}$ (Cross-validation coefficient of determination ${(R}_{cv}^{2})$ for Y-Randomization test) has been established to determine the validity of the model and whether it is the result of random correlation. The evaluation involves using the following four specific inequalities to check the accuracy of the models that were developed [52]:

$Q_{yrand}^{2}≺0.2$: No possibility of a relationship by chance

$0.2{≺Q}_{yrand}^{2}≺0.3$: Negligible probability of a relationship by chance

$0.3{≺Q}_{yrand}^{2}≺0.4$: Not a significant random relationship

$Q_{yrand}^{2}≻0.4$: Seem to be correlated by chance

#### 2.2.5 Applicability domain

To specify the region of chemical space where the predictions of anti-cervical activities are reliable, the applicability domain(AD) of the best model was determined using the leverage technique (William's diagram) [53,54]. For this purpose, the most common method is to evaluate the leverage values h_i_ for each individual compound as given in Eq. (6). Accordingly, a compound is considered to be excluded from the domain of applicability when its leverage h_i_ exceeds the predefined alert threshold h* as defined in Eq. (7).

(Eq. 6) $$h_{i}=x_{i}^{T}{\left(X^{T}X\right)}^{-1}x_{i}$$

(Eq. 7) $$h^{*}=3\times \frac{\left(K+1\right)}{n}$$

where i ranges from 1 to n, and x_i_ designates a vector characterizing the chemical compound to be identified, while X represents a matrix encapsulating the k values of the descriptors' model for the n compounds in the training set.

### 2.3 Molecular docking simulation

In order to obtain the kinds of interactions between the ligands identified and the targeted active site responsible for inhibiting cervical cancer, molecular docking modelling was used. Specifically, the most active molecule 34 (Mol. 34) and the least active molecule 21 (Mol. 21) as well as the designed drug-candidates, were docked to the aromatase active site (PDB: 3EQM) [55]. Using Discovery Studio software, we firstly purified the receptors of each protein of interest by removing all original ligands, water molecules and other non-protein components [56,57]. Then, the ligand-protein interactions were analyzed using Auto-Dock version 1.5.6 [58]. Accordingly, the dimensions of the grid were determined via AUTOGRID and the established interactions were examined in more detail in the two-dimensional and three-dimensional representations using Discovery Studio software. To check the validity of the docking method, the co-crystallized ligand was re-docked and the root mean square deviation (RMSD), which must be less than 2 Å, was estimated [59].

### 2.4 Pharmacophore analysis

The integration of pharmacophore modelling with molecular docking studies contributes to the discovery of new drug-candidates that have some essential features in common with known active molecules [60,61]. Therefore, the representation of essential features through pharmacophore models is closely linked to molecule selection as well as the validation of molecular docking interactions. In this contribution study, we used the UNITY module of SYBYL-X 2.1 to build 3D pharmacophore models for the cervical cancer inhibitors studied. The conformers were generated using the Genetic Algorithm (GA) method and the GALAHAD (Linear Algorithm for Hypermolecular Alignment of Data Sets) platforms [62,63]. For this, the features of the pharmacophore were developed by flexibly superimposing the compounds in the training set to obtain hypermolecular alignments and then validated using the molecules in the test set. Finally, the best pharmacophore model may be used to validate the molecular docking results of new drug-candidates [64,65].

### 2.5 Pharmacokinetics and drug likeness investigations

Since it is generally recognized in practice, effective and useful therapeutic agents are characterized by their pharmacodynamic and pharmacokinetic properties which include important parameters such as high therapeutic potential, affinity, acceptable levels of toxicity and selectivity for the specific molecular target [66]. As part of this research contribution, the assessment of chemical absorption, distribution, metabolism, excretion and toxicity (ADMET), as well as drug similarity characteristics, was performed using online tools such as pkCSM and SwissADME. For this purpose, in silico predictions were included concerning the caco-2 permeability assay (apparent permeability coefficient (Papp)), intestinal absorption, the blood-brain barrier and penetration into the central nervous system, biotransformation, clearance and the AMES test of drug-candidates [[66], [67], [68]].

## 3 Results and discussion

### 3.1 2D-QSAR models

#### 3.1.1 MLR models

Using pertinent molecular descriptors and experimental values of cervical cancer activity, a number of models have been developed (Table S_2_). Any model that does not comply with the Organization for Economic Cooperation and Development's (OECD) as well as Golbraikh and Tropsha's criteria was rejected [69]. The equation of the best model built using the MLR approach is given in Eq. (8):

(Eq. 8) **pIC**_**50**_ = **0.481** + 0.063 × **NHA** + 0.334 × **NHD** + 0.092 × **DM** + 1.510 × **Q** + 1.019 × **Q**_**1**_ - 15.868 × **E**_**HOMO-1**_ + 0.922 × **SB**

The variance inflation factor (VIF), denoted VIF = 1/1-$r^{2}$ (where r^2^ represents the correlation coefficient of multicollinearity), was applied to choose the most suitable descriptors for each model generated. Thus, only descriptors with VIF values below 5 were selected (Table 3) [70].

**Table 3:** Chosen descriptors' VIF values for the best MLR model.

Model's descriptors	NHA	NHD	DM	Q	Q_1_	E_HOMO-1_	SB
**VIF**	1.418	2.407	1.571	1.350	1.393	3.037	2.616

**Fig. 1:**
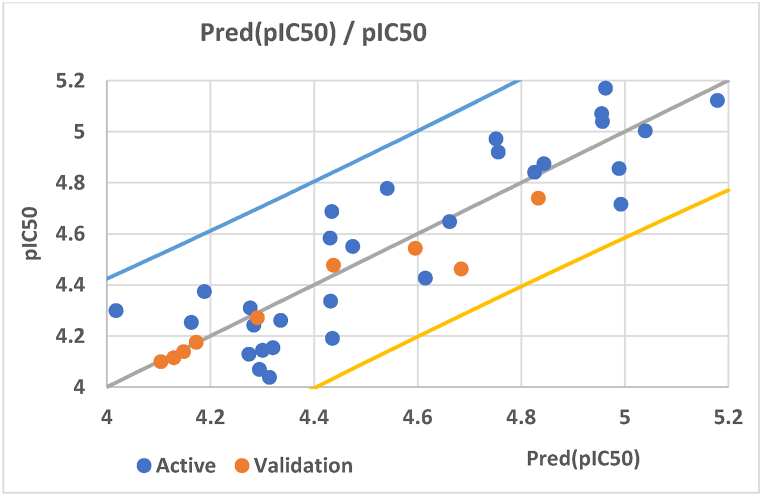
Correlation diagram showing computed vs experimental pIC_50_ of training and test sets.

According to Table 3, all descriptors of the best model had VIF values below 5, confirming the absence of multicollinearity. The reliability of this model was then confirmed on the basis of its stability, robustness and predictability. To assess the stability of the best multiple linear regression (MLR) model, 100 separate permutations of compounds in the training set were used in independent tests, resulting in 100 new models with varying $R_{yrand}$ , $R_{yrand}^{2}$, $Q_{yrand}^{2}$ and ${cR}_{r}^{2}$ values. The robustness of the best model was affirmed by its high coefficient of determination ($R^{2}$ = 0.779) for the relationship between descriptors and activities, complemented by the adjusted coefficient of determination ($R_{adj}^{2}=$ 0.709), highlighting the real impact of selected descriptors on pIC_50_. The coefficient of determination of Leave-One-Out Cross-Validation ($R_{CV}^{2}$ = 0.586) emphasizes the strong internal predictive capacity of the model, while the considerable value of the coefficient of determination of external validation (${\;R}_{test}^{2}$ = 0.658) demonstrates its significant prediction ability for the new proposed molecules. Furthermore, VIF (≤5), $R_{yrand}^{2}\left(0.238\right)$, $Q_{Yrand}^{2}$ (−0.432), ${cR}_{p}^{2}\;($ 0.658) and MSE (0.037) values collectively confirm the model's ability to predict the pIC_50_ values of new drug candidates from the quinazoline and thioquinazolinone derivatives studied. Fig. 1 displays the correlation diagram between the predicted pIC_50_ values and their corresponding experimental values for both the training and test sets of the MLR model.

As illustrated in Fig. 1, there is a significant correlation between the observed and predicted pIC_50_ values, highlighting the robust ability of the model to predict the pIC_50_ values of new compounds specifically designed to treat cervical cancer. This predictive ability may be based on the well-interpretation of descriptors selected by the model.

#### 3.1.2 Interpretation of selected descriptors

**Fig. 2:**
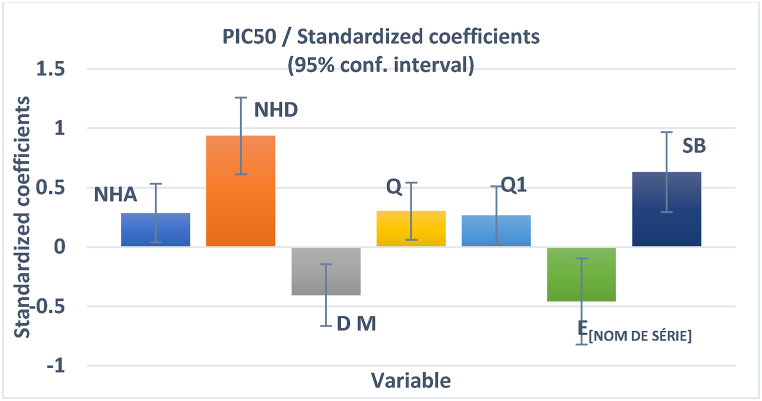
Characterization modeling with normalized coefficients.

According to Eq. (8), the best MLR model is characterized by geometric, electronic, and steric descriptors including NHA, NHD, DM, Q, Q1, HOMO-1, and SB. Similarly, to parameters of Student's t-test, the coefficient normalization diagram (Fig. 2) provides an overview of the importance and impact of each descriptor on the predicted anti-cervical cancer activity.

Fig. 2 clearly shows that NHD and stretch-bonds energy (SB) are the first and second most significant factors in the model, with a positive sign in Eq. (8), suggesting that higher values of these parameters may increase the anti-cervical cancer activity of the molecules under study. The third and fourth critical parameters of the model, both with a negative sign in Eq. (8), are the electronic descriptors (HOMO-1 energy and the dipole moment (DM)) which have a suppressive effect on this activity. In this case, the HOMO-1 orbital has a low value, indicating that it will be difficult to remove an electron from the molecule and that the inhibition of activity against cervical cancer could be inversely proportional to this property. Moreover, the improvement in this activity is not only attributable to the reduction in dipole moment, but also to the reduction in asymmetric dispersion of charges. However, NHA, Q and Q_1_ charges are all positively correlated with the biological activity studied. According to these results, the NHD and NHA, the electrostatic interactions (DM, Q and Q_1_) and SB (vibration mode) all play a major role in the anti-cervical cancer activity of the investigated compounds.

**Fig. 3:**
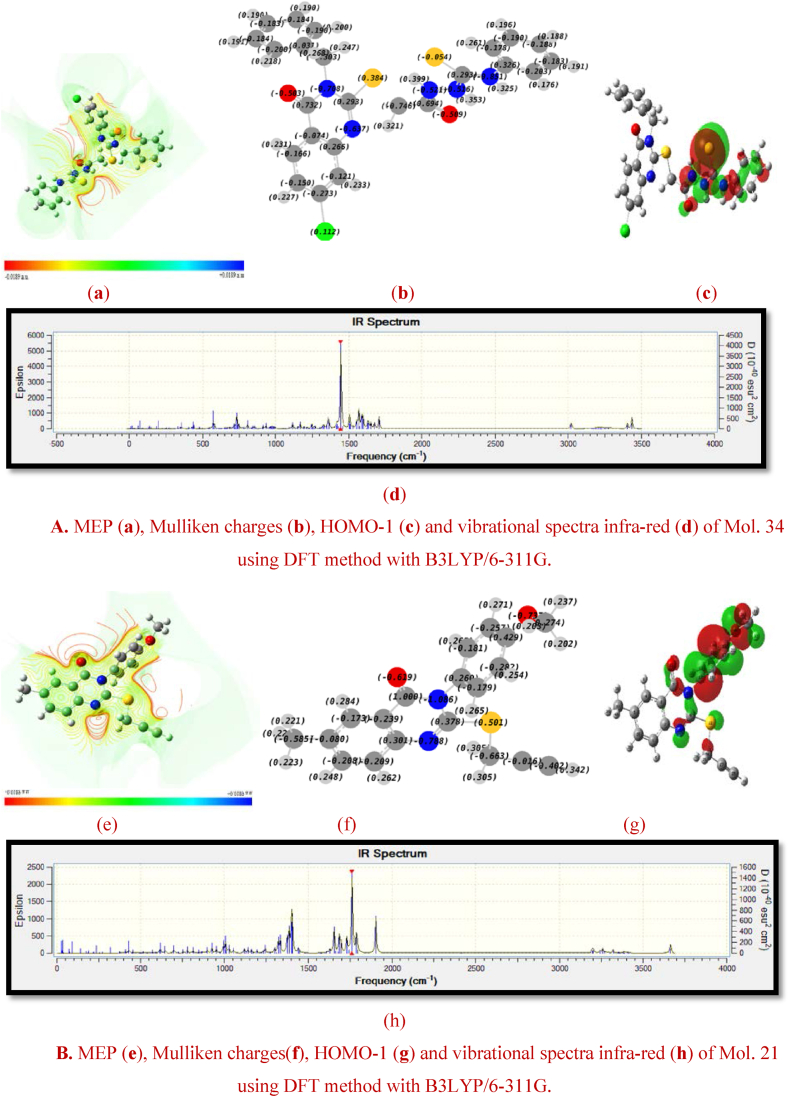
AMEP (**a**), Mulliken charges (**b**), HOMO-1 (**c**) and vibrational spectra infra-red (**d**) of Mol. 34 using DFT method with B3LYP/6-311G. B. MEP (**e**), Mulliken charges (**f**), HOMO-1 (**g**) and vibrational spectra infra-red (**h**) of Mol. 21 using DFT method with B3LYP/6-311G.

Certainly, the electronegativity of molecules may be a determining characteristic of their chemical interactions. In addition, the polarity of a chemical bond is directly proportional to the electronegativity of the atoms being bonded. In cases where the bonded atoms have unequal electronegativity, the bond aligns itself in the direction of the most electronegative atom. However, the resultant force generated by the spatial distribution of charges within the system mediates these interactions. Accordingly, dipole moment (DM) which is represented as the product of the partial charges (Q) of the atoms and the bond separation (r) may become a valuable parameter in this context. More specifically, its value may also provide an insight into the distribution of charges, which explains the nature of the interactions between the different chemical species, including electrostatic and dipole-dipole attractions. In fact, molecular structures whose centers of inertia of positive and negative charges are almost identical have a zero permanent dipole moment, resulting in minimal interactions with other non-polar substances. Nevertheless, it's clear that the variation in the permanent dipole moment, influenced by chemical disorder, depends on the atomic arrangements within the molecule. Additional information about the influence of these arrangements on the charge distribution, and consequently on the permanent dipole moment, may be obtained by calculating the isosurfaces of the molecular electrostatic potential (MEP). This means that the MEP may be an invaluable parameter for research into the chemical reactivity or bioactivity of an anti-cervical cancer compound [[71], [72], [73]]. In fact, the spatial distribution of the electrostatic potential is a main factor in determining whether a molecular interaction is initiated by an electrophilic or nucleophilic agent (particularly intermolecular interactions). In addition, the three-dimensional distribution of the electrostatic potential is of great importance, particularly in the context of the binding of a substrate to the active site of the protein under study. It should be noted that chemical disorder (substituent groups) can exert a significant influence on the vibrational modes of molecules, particularly with regard to stretch-bonds. This influence may be due to a reduction in the energy gap between the frontier orbitals, accompanied by a redistribution of the charge density [74,75]. The energy level of HOMO-1, which measures a molecule's ability to donate electrons, is a key frontier molecular orbital defining electrical properties in quantum chemistry and having a significant impact on p-type charge transport. In addition, it is essential to recognize that covalent bonds in molecules are flexible, allowing them to be stretched and twisted. At standard room temperature, these bonds exhibit different vibrational modes with vibrational energies classified into quantum levels similar to electronic states. It is crucial to emphasize that the absorption of infrared photons leads to transitions between these vibrational energy levels. In particular, the energy required to stretch (or compress) a bond is significantly higher than that required to bend it. Therefore, it is important to note that the presence of substitution groups has a substantial impact on the infrared stretching frequencies of various bioactive molecules. Fig. 3A and B provide a visual representation of the molecular electrostatic potential (MEP) surface, the Mulliken charge values for the component atoms (Qi), the molecular frontier orbitals (HOMO-1) and the infrared vibrational spectra of the Mol. 34 and Mol. 21.

From Fig. 3A (a) and Fig. 3B (e) of MEP, blue indicates electropositive regions, green indicates neutral zones, and red indicates electronegative regions. Accordingly, the most positively charged portions of the MEP surface are hydrogen and carbon atoms which are predicted to be attractive places for nucleophilic interaction or hydrogen bond donor. The carbonyl oxygen O and atom S are the most negatively charged portions of the molecule, making them the primary locations for electrophilic interaction and hydrogen bond acceptors. By comparing the Mulliken charge values for the constituent atoms of Mol. 34 and Mol. 21 (Fig. 3A (b) and Fig. 3B (f)), it becomes clear that electronic charges play a crucial role in establishing the binding ability of anti-cervical cancer inhibitors. More specifically, the highest positive charges are found in hydrogen atoms bonded to oxygen and nitrogen atoms because of their high electronegativity. Conversely, among the positively charged carbon atoms, those linked to O and S atoms. Consequently, the spatial distribution of HOMO-1 orbitals, which is proportional to the electron density as well as its energy value, varies considerably between the benzene ring of Mol. 21 and its substituted group (Fig. 3A (c) and Fig. 3B (g)). In comparison, an examination of the most active molecule reveals that the HOMO-1 contour surfaces are mainly concentrated on the C=S bond, with an accumulation of electronic clusters around S atom, indicating its involvement in charge transfer processes. This phenomenon may be due to the presence of more electronegative elements that also interact as hydrogen bond donors and acceptors. Accordingly, structural modifications from the Mol. 21 to Mol. 34 led to changes in electronic states, characterized by a reduction in dipole moment (DM) via a symmetrical charge distribution, an increase in HOMO-1 energy and an increase in the energy of stretch-bonds (stretch vibrations). The fact that replacing the benzene ring with a halogen atom (Cl) may well induce a significant polarization in the Mol. 34. This polarization increases the potential for symmetrical charge distribution, thereby increasing electrostatic interactions while minimizing the DM. In particular, this modification leads to the generation of new vibrational modes in the infrared (IR) spectrum, characterized by very different stretching vibrations, as shown in Fig. 3A (d) and Fig. 3B (h). In fact, the substitution of a carbon-carbon triple bond by 2-acetyl-N-phenylhydrazine-1-carbothioamide in the external chain may increase not only the number of hydrogen bonds, but also the vibrational modes that are related to stretch bonds. Thus, the IR absorption associated with the C=S stretching vibration has been generally observed in the 800 to 1500 cm-^1^ frequency range because the precise position of this absorption region depends on the specific chemical environment and the atoms bonded to the thiocarbonyl carbon. In cases where nitrogen atoms are bonded to thiocarbonyl derivatives, the IR spectra show three distinct coupled C=S vibrations, appearing in the frequency ranges 1570-1395 cm-^1^, 1420-1260 cm-^1^, and 1140-700 cm-^1^.

These results highlight the primordial importance of the halogen atom (Cl) and the sulfur atom in anti-cervical cancer derivatives, as well as the importance of the most vibrational groups wherein the hydrogen is directly bonded to highly electronegative elements (NHD and NHA), resulting in a remarkable positivity of the hydrogen charges that are favorable for electrostatic and hydrogen bond interactions. Similarly, despite the need for strategic structural modifications based on structural and electronic factors, the potential for designing a new anti-cancer agent should be enhanced by the predictive power of the model generated. However, it is important to admit that the study's reliance on 2D-QSAR modelling has inherent limitations, particularly when it mainly accounts for molecular properties and activities on the basis of two-dimensional representations. Moreover, anti-cervical cancer compounds and their interactions with biological targets occur in three dimensions, whereas a 2D model cannot take this type of interactions into account. Therefore, the next steps could be to investigate the visual characteristics of molecular interactions in more detail, using 3D-QSAR (CoMFA and CoMSIA fields), molecular docking and pharmacophore modeling.

### 3.2 3D-QSAR models

**Fig. 4:**
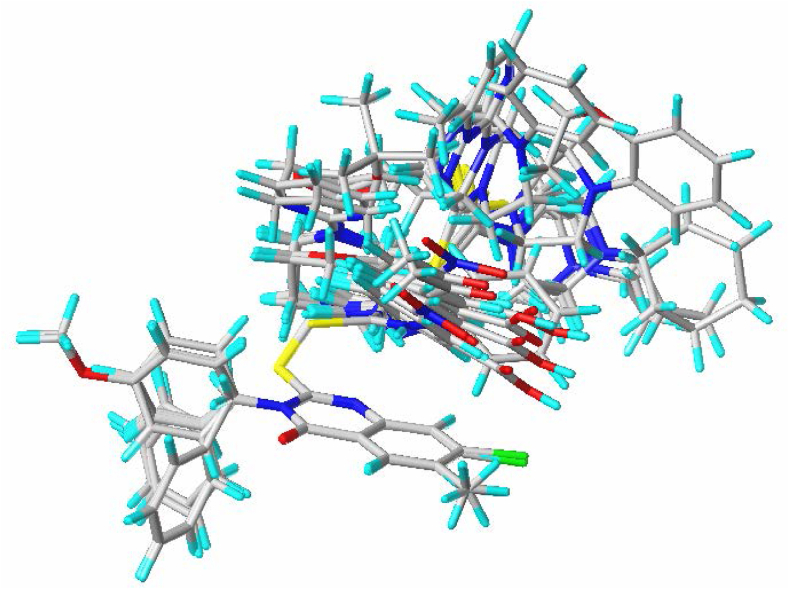
Molecular alignment of the 39 investigated derivatives using Mol. 34 as template.

Mol. 34, which has the most significant biological activity (pIC_50_ = 5.170), was chosen as the reference compound to align the molecules of the same dataset used in the 2D-QSAR study and to build the essential contour maps for the 3D-QSAR models (Fig. 4).

After molecular alignment, nine distinct combinations of CoMFA and CoMSIA fields were used to build the 3D-QSAR models employing the PLS approach. The relevant parameters for these models are detailed in Table 4. Accordingly, the selection of the best model was based on several criteria, including the highest $R_{CV}^{2}$ (0.619) and $R^{2}$ (0.951) values of the model, the lowest values for the number of principal components (N = 3) and the standard error of estimation (SEE = 0.113) as well as the significant value of F _test_ (66.200).

**Table 4:** Measures of statistical significance for the CoMFA and CoMSIA models.

Model	$R_{CV}^{2}$	N	*R*^2^	SEE	F
CoMFA	0.489	3	0.884	0.129	66.168
COMSIA/SEH	0.400	4	0.890	0.128	50.401
COMSIA/EDA	0.613	4	0.860	0.144	58.348
COMSIA/EHA	0.589	3	0.855	0.144	50.952
COMSIA/SEHD	0.445	5	0.940	0.096	75.648
COMSIA/SEHA	0.581	3	0.860	0.142	53.078
COMSIA/SEDA	0.619	3	0.951	0.113	66.200
COMSIA/EHDA	0.588	3	0.863	0.140	54.557
COMSIA/SEHDA	0.569	3	0.870	0.137	57.859

**Fig. 5:**
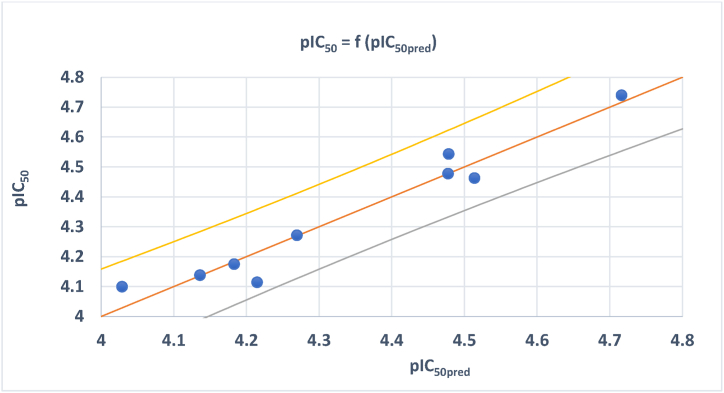
Graph comparing experimental pIC_50_ to those predicted by CoMSIA/SEDA model for test set.

As a result, the best model (CoMSIA/SEDA) was determined by including the steric (S), electrostatic (E) and hydrogen bonding donor (D) and acceptor (A) fields. This model may also be able to give the highest external coefficient of determination as well as its predictive power. Fig. 5 illustrates visually the relationship between observed and predicted activities for a set of external test molecules.

Examining this graph more carefully, it is apparent that the slope is approaching 1, while the y-intercept is close to 0. These observations confirm the model's ability to predict the pIC_50_ of the molecules tested via external validation. Before applying the selected model to the prediction of new drug-candidates with a high degree of confidence, it also needs to be validated according to the criteria defined by Golbraikh and Tropsha (Table 5).

**Table 5:** Statistical criteria for CoMSIA/SEDA model validation.

Statistical parameter	Model's score	Threshold	Validation
$R_{\text{test}}^{2}$	0.946	>0.600	Yes
${R_{0}}^{2}$ ; The predicted versus observed zero intercept correlation coefficient	0.946	>0.600	Yes
${R_{0}^{\prime }}^{2}$ ; The observed versus predicted zero intercept correlation coefficient	0.946	>0.600	Yes
$|{R_{0}}^{2}-{R_{0}^{\prime }}^{2}|$	0.000	Under 0.300	Yes
$\frac{R^{2}-R_{0}^{2}}{R^{2}}$	0.005	Under 0.100	Yes
$\frac{R^{2}-R_{0}^{\prime 2}}{R^{2}}$	0.005	Under 0.100	Yes
K; Zero intercept slope of test set predicted vs. observed activity	0.999	0.850≺K≺1.150	Yes
$K^{\prime }$ ; Zero intercept slope of test set observed vs. predicted activity	1.001	$0.850≺K^{\prime }≺1.150$	Yes
$R_{m}^{\prime 2}=R^{2}\left(1-\sqrt{(R^{2}-}R_{0}^{\prime 2}\right)$	0.884	>0.600	Yes
$R_{m}^{\prime 2}=R^{2}\left(1-\sqrt{(R^{2}-}R_{0}^{\prime 2}\right)$	0.884	>0.600	Yes

From the results of Table 5, the proposed CoMSIA/SEDA model can be classified as validated, provided that it is not considered as random modelling. To check the robustness of this model, Y randomization tests were performed using five random rearrangements of pIC_50_ (Table 6).

**Table 6:** Y-Randomization statistical parameters.

Iteration	COMSIA/SEDA		
	$Q_{\text{yrand}}^{2}$	$R_{\text{yrand}}^{2}$	${\text{cR}}_{p}^{2}$
1	0.358	0.747	0.571
2	0.267	0.760	0.588
3	0.186	0.699	0.515
4	0.186	0.721	0.539
5	0.331	0.698	0.514

The values of $Q_{yrand}^{2}$, $R_{yrand}^{2}$ and ${cR}_{p}^{2}$ indicate that a random correlation is improbable, confirming the reliability of the CoMSIA/SEDA model which may be used to predict new anti-cervical cancer agents.

**Fig. 6:**
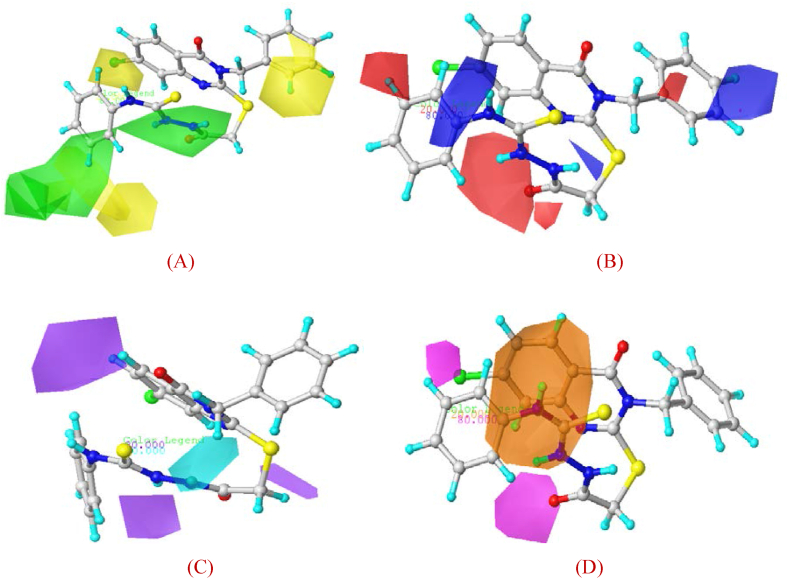
Steric (**A**), Electrostatic (**B**), HBD (**C**) and HBA (**D**) contour maps of Mol. 34.

For a more detailed understanding of the information contained in the best 3D-QSAR model (CoMSIA/SEDA), contour maps illustrating the steric, electrostatic, HBD and HBA fields are shown in Fig. 6 (A-D). Accordingly, green contour maps represent the sterically advantageous regions with increased bulk, whereas yellow contour maps represent the sterically unfavorable regions (Fig. 6 (A)). Areas of high positive electrostatic preference are delineated by blue contour maps, whereas areas of high negative electrostatic preference are delineated by red contours in the electrostatic field (Fig. 6 (B)). The HBD field is shown in cyan and purple to indicate regions where hydrogen-bond donor characteristics are preferred and unfavorable, respectively (Fig. 6 (C)). Magenta contour maps for HBA groups enhance activity, whereas orange contour maps indicate the unfavorable region (Fig. 6 (D)).

According to Fig. 6 (A-D), the green contours around the benzene adjacent to the substituted group (R) and the external chain (2-acetyl-N-phenylhydrazine-1-carbothioamide) indicate the potential for enhanced anti-cervical cancer activity through steric group substitution in these areas. On the other hand, the regions between Cl and H, as indicated by the yellow contours around benzene, are susceptible to decreased this activity when occupied by bulky groups. The red contour maps surrounding the areas of the modified group (R) and the external chain (2-acetyl-N-phenylhydrazine-1-carbothioamide) suggest that the addition of high electronegativity groups or atoms can potentially increase the activity. The blue contour maps surrounding the hydrogen atom of the benzene group suggests that the incorporation of highly electropositive groups or atoms in this region could increase the activity against cervical cancer. The presence of cyan contour maps around the external chain (2-acetyl-N-phenylhydrazine-1-carbothioamide) and the benzene at the junction of the substituted group (R) indicates the potential improvement in activity with HBD groups. Conversely, substituting HBD in areas indicated by purple outlines is likely to reduce activity. The magenta contour maps, with a contribution of 80 %, highlight the importance of Cl, O, N and the modified group (R) in improving activity by replacing an acceptor of hydrogen bonding. While, the orange contour maps indicate unfavorable areas for HBA. These results may be justified by comparing the Mol. 34, containing a halogen atom (Cl) and HBD/HBA groups, with Mol. 21 containing –CH_3_ and –OCH_3_ groups.

Based on the information from the contour maps and the significance of the field fractions presented in Table 7, it is apparent that an increase in electrostatic interactions (constituting 30.2 % of the contribution), an increase in the number of HBA (28.8 %) and an increase in the number of HBD (28.1 %) have a significant favorable impact on anti-cervical cancer activity. Conversely, an increase in steric interactions (12.9 %) is associated with a reduction in this activity. To improve activity of the new ant-cervical cancer derivatives, a strategic approach could be to replace the modified group (R) of Mol. 34 with a less sterically challenging group characterized by increased electrostatic potential and a significant number of HBD/HBA (for example, carboxyl and amide groups containing –OH and –NH_2_).

**Table 7:** CoMSIA/SEDA fraction analysis.

COMSIA Fields	Norm. Coeff.	Fraction
COMSIA_ Steric (S)	0.449	0.129
COMSIA_Electrostatic (E)	1.047	0.302
COMSIA_Donor (D)	0.977	0.281
COMSIA_Acceptor (A)	0.999	0.288

In order to be used reliably, the best models were tested to predict the activities of the molecules in the learning and test sets studied. The pIC50 values predicted by the CoMSIA/SEDA model and the MLR model are presented in Table S_3_. By comparing these values with the experimental values, it is clear that the MLR and CoMSIA models can be considered robust and predictive. As a result, these models enable the biological activity of new drug candidates to be predicted with a high degree of accuracy provided they belong to the same domain of applicability.

### 3.3 Applicability domain

**Fig. 7:**
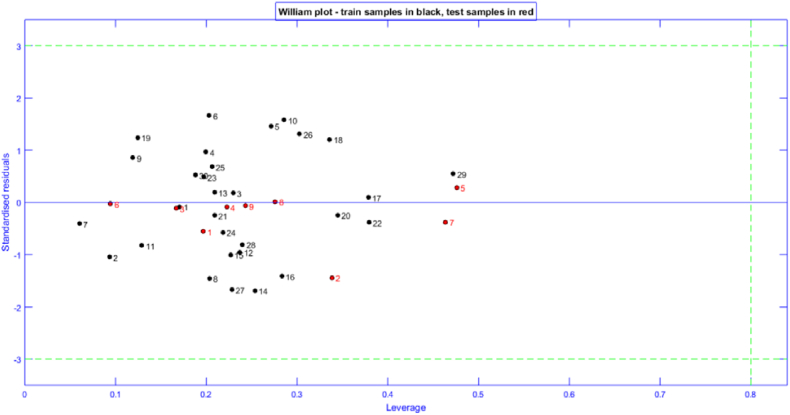
Applicability domain (AD) of the best model.

The applicability domain (AD) of the best model was evaluated using a leverage analysis based on the William's diagram. Fig. 7 illustrates the results, with all compounds in the training and test sets having values of leverage h_i_ below the alert threshold (h* = 0.80), verifying their including within the applicability domain. This confirms that the model can reliably guide the design of new compounds with improved anti-cervical cancer activities limited to this AD.

### 3.4 Design of novel drug-candidates

**Fig. 8:**
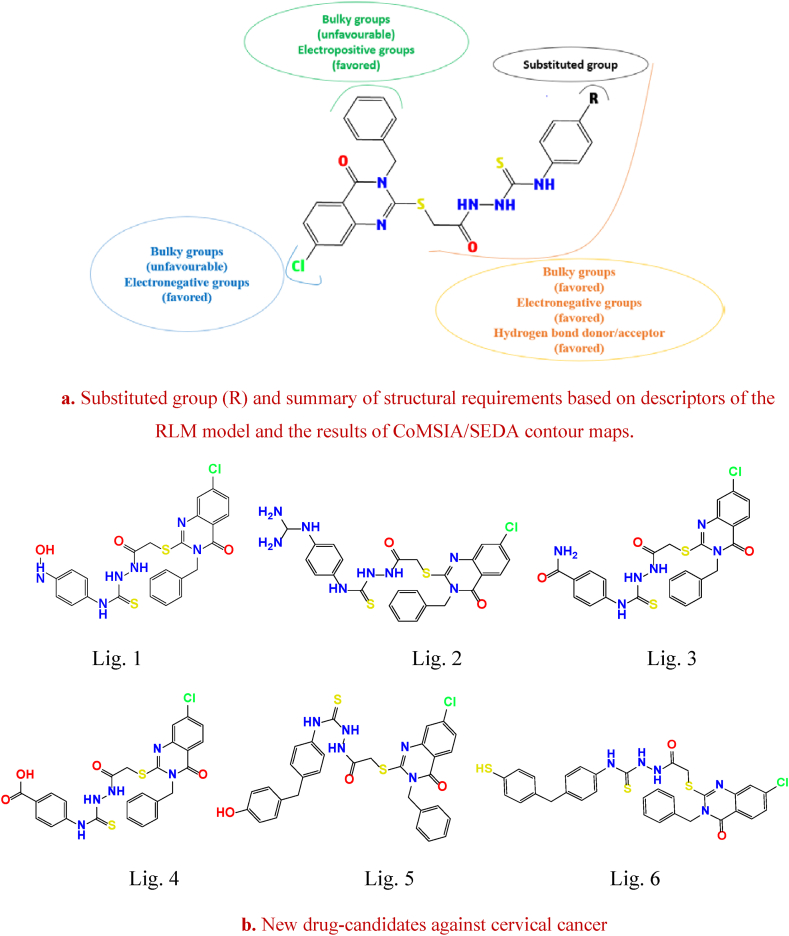
Substituted group (R) and summary of structural requirements based on descriptors of the RLM model and the results of CoMSIA/SEDA contour maps. New drug-candidates against cervical cancer.

Exploiting the predictive ability of robust 2D/3D QSAR models, six new ligands (Lig. 1, Lig. 2, Lig. 3, Lig. 4, Lig. 5, and Lig. 6) have been designed with improved activities against cervical cancer by modifying the molecular structure (substitutes group (R)) of Mol. 34 and based on virtual fragments of Zinc database (Fig. 8a and Fig. 8b) [76,77].

The newly designed compounds (Lig.1 - Lig.6) were optimized and aligned in a similar way to the previously studied dataset, in order to calculate their descriptors and predict their biological activity, using Mol. 34 as a reference. In addition, their similarity to drugs and their AD were also evaluated. Table 8 summarizes the predicted descriptors using the same methodology as previously, the biological activities predicted using 2D/3D QSAR models, and the determination of the leverage effect (hi) via Eq. (6) as well as an assessment of the synthetic accessibility (SA) [78] and Lipinski criteria [79] using the SwissADME web platform [42].

**Table 8:** Descriptor values, pIC50 predictions, hi values, SA, and Lipinski validation for novel drug-candidates.

Ligand	NHA	NHD	D M	Q	Q_1_	HOMO-1	SB	pIC_50_pred (MLR)	pIC_50_pred (CoMSIA)	hi	AD	SA	Lipinsky validation
**Lig. 1**	5	5	4.971	0.1986	−0.290	−0.228	0.148	5.368	5.201	1.047	Outside	3.650	Yes
**Lig. 2**	6	6	8.766	0.001	−0.299	−0.214	0.207	5.332	5.128	1.535	Outside	3.850	No
**Lig. 3**	4	4	4.782	0.211	−0.314	−0.233	0.223	5.531	5.306	0.793	Inside	3.650	Yes
**Lig. 4**	4	4	3.043	0.092	−0.289	−0.237	0.163	5.344	5. 180	0.478	Inside	3.600	Yes
**Lig. 5**	4	4	3.412	0.001	−0.299	−0.225	0.152	5.160	5.091	0.461	Inside	4.020	No
**Lig. 6**	3	4	3.057	0.045	−0.318	−0.234	0.153	5.324	5.093	0.486	Inside	4.040	No

**Fig. 9:**
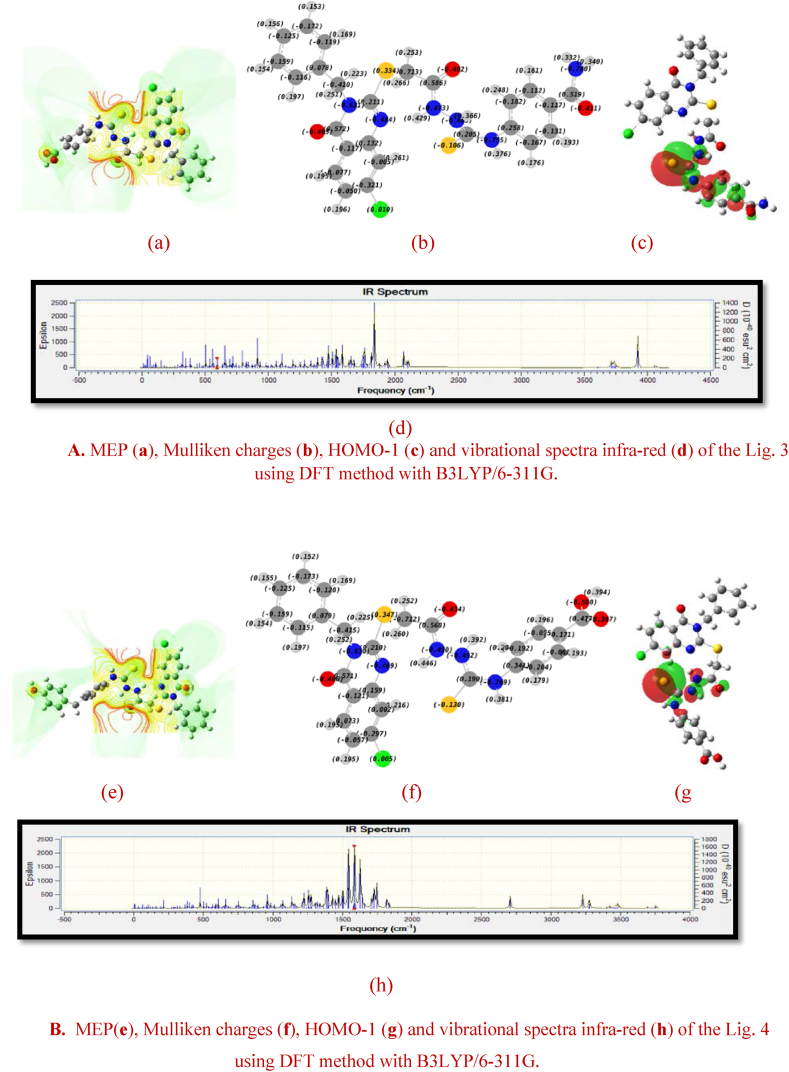
A. MEP (**a**), Mulliken charges (**b**), HOMO-1 (**c**) and vibrational spectra infra-red (**d**) of the Lig. 3 using DFT method with B3LYP/6-311G. B. MEP(**e**), Mulliken charges (**f**), HOMO-1 (**g**) and vibrational spectra infra-red (**h**) of the Lig. 4 using DFT method with B3LYP/6-311G.

The results shown in Table 8 demonstrates that the designed ligands (Lig. 1 - Lig. 6) exhibit more activity than Mol. 34. In addition, we find that the S.A values of these drug-candidates range from 3.600 to 4.040 indicating that these values are far below 10 and close to 1 which make the synthesis of these molecules relatively possible. Based on the criteria provided by AD and Lipinski validation, only 2 drug-candidates (Lig. 3 and Lig. 4) were selected. Fig. 9A (a-d) and Fig. 9B (e-h) depict the mapping of MEP, Mulliken charges, HOMO-1 and vibrational spectra infra-red of the newly selected ligands (Lig. 3 and Lig. 4).

Analyzing Fig. 9A (a-d) and Fig. 9B (e-h), and comparing them with Fig. 3A above, an intriguing observation can be made. When the substituted hydrogen atom (R=H) of Mol. 34 (containing S and Cl atoms) is replaced by an amide group or a carboxylic acid group (electronegative and HBD/HBA), a number of effects have been shown to improve anti-cervical cancer activity. As a result of these substitutions, the vibrational modes (including the SB descriptor) increase with increased charge dispersion (Mulliken charges) symmetrically to the center of inertia of Lig. 3 and Lig. 4, as well as with a reduction in dipole moment (DM) and an increase in electrostatic interactions (MEP), and in particular with a smaller HOMO-1 molecular orbital. There were also attempts to examine the difference between the vibrational spectra of Mol. 34 and the designed compounds. The absorption of infrared (IR) light in the bands (1600-1800 cm^−1^), (1470-1570 cm^−1^), (1250-1350 cm-^1^) and (3300-3500 cm^−1^) correlates well with the characteristic groups of the Lig. 3 and Lig. 4. When the carbonic amide signal (Lig. 3) disappears, it is replaced by a peak characteristic of carboxylic acid (Lig. 4) because the production of hydrogen bonds often generates a vibrational stretching frequency. When HBD and HBA are in the same group (Lig. 3 and Lig. 4), the accumulation of charges is greater than in systems where the donors and acceptors are not the same (Mol. 34), resulting in stronger electrostatic interactions in parallel with the favorable hydrogen bond interactions. In this case, the ability to form hydrogen bonds may be proportional to the acidity of the carboxyl group and also depends on the length and position of the chain under study containing the group to be modified (R). We may therefore deduce that steric, electrostatic and hydrogen bonds are the basis of the specific interaction between cervical cancer inhibitors and the active site of the protein studied. Finally, it is important to note that these results are consistent with the interpretations obtained from the descriptors of the RLM and CoMSIA contour maps.

Despite the fact that 3D QSAR approaches have provided significant information on 3D interactions, they have certain limitations in terms of precision and ligand-protein interactions. To overcome these deficiencies, molecular docking offers the possibility of simulating three-dimensional interactions, taking into account the mechanism and types of interactions required. This approach will improve the reliability of predictions concerning ligand-protein complexes, optimize the design of new specifically targeted compounds, and validate the previous results.

### 3.5 Molecular docking study

**Fig. 10:**
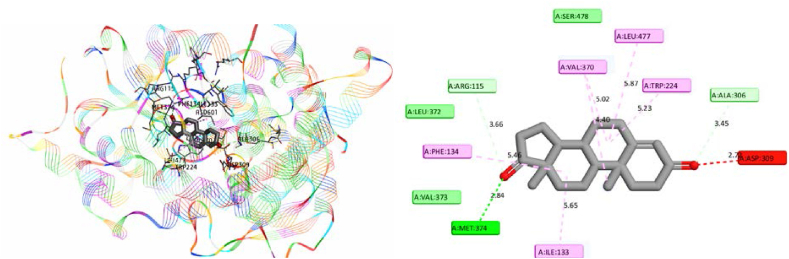
Aromatase - Androstenedione interactions.

Molecular docking is an essential technique in drug development, contributing mostly in discovery of essential features required for inhibition of the disease studied by exploring the interactions between special proteins and their corresponding ligands. In this study, we adopted the protein aromatase (PDB ID: 3EQM) for docking with the original substrate 4-androstene-3-17-dione (Androstenedione) as co-ligand. The main objective was to elucidate the binding interactions between quinazoline and thioquinazolinone derivatives and the identified active site of aromatase enzyme in order to design new potent drug-candidates [28]. Firstly, the visualization of active site identified specific residues namely Arg 115, Val370, Leu 477, Trp224, Ala 306, Asp 309, Ile 133, Met 374, Phe 134, Ser 478, Val 373 and Leu 372 as essential for cervical cancer inhibition processes (Fig. 10). These key amino acids may represent an essential part of the next molecular docking studies.

**Fig. 11:**
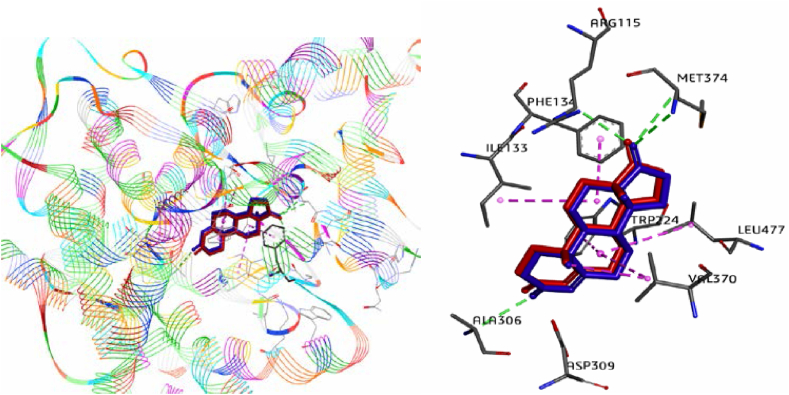
Redocking of Androstenedione with aromatase (blue = co-ligand and red = redocked ligand). (For interpretation of the references to colour in this figure legend, the reader is referred to the Web version of this article.)

To validate the molecular docking, the protein's co-crystallized ligand (Androstenedione) was re-docked at the same active site. For this, grid maps were made with default grid space sizes of 0.375 Å and 60*60*60 Å^3^ in all directions. The coordinates x = 85.381 Å, y = 54.088 Å and z = 45.807 Å define the middle box of the grid that corresponds to the location of the ligand in the active site. Following the finishing of the docking process, more detailed exploration of the ligand interactions linked to the target protein was carried out via 2D and 3D visualizations using Discovery Studio software as shown in Fig. 11. As a result of the redocking procedure, the co-crystallized ligand and the redocked ligand were superimposed with RMSD = 0.378 Å.

**Fig. 12:**
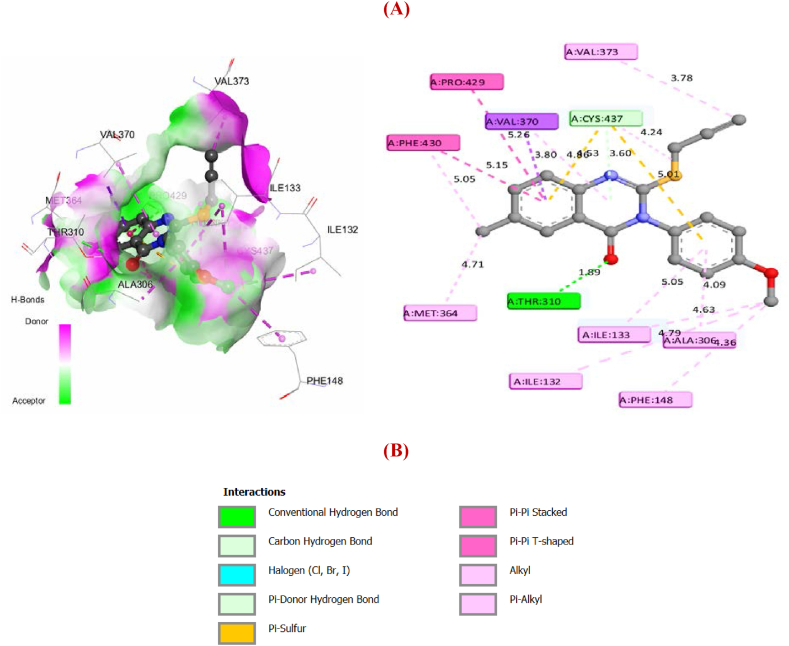
3D and 2D interactions of Mol. 34 (**A**) and Mol. 21 (B) with aromatase enzyme.

While the molecular docking was successfully validated, priority was given to docking Mol. 34 and Mol. 21 in order to extract key information that could be exploited to investigate new drug-candidates. As shown in Fig. 12 (A, B), 2D and 3D interactions of this process were explored.

**Fig. 13:**
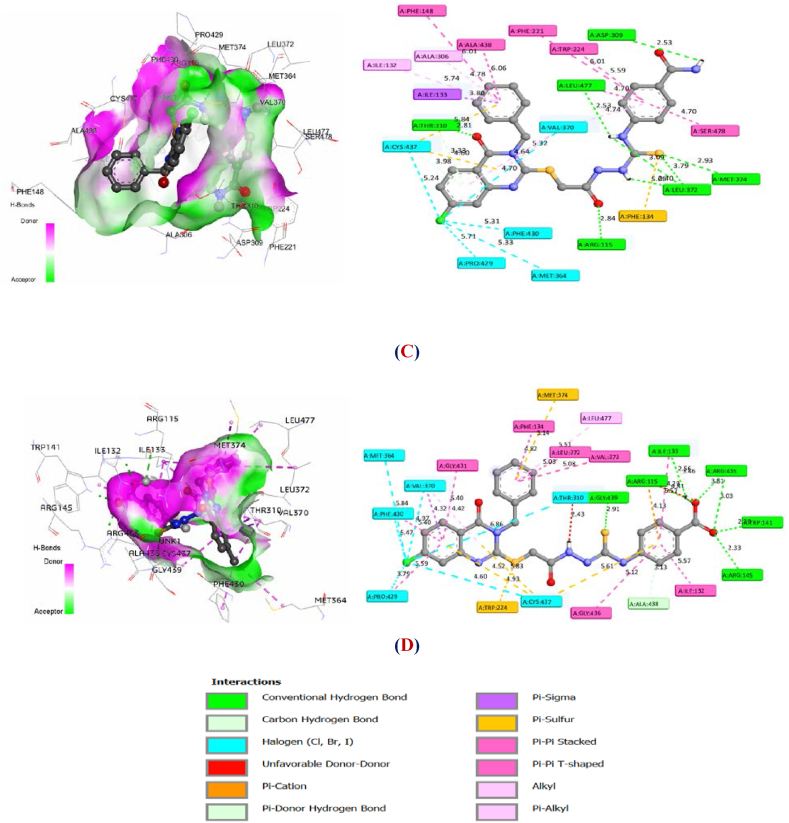
3D and 2D interactions of Lig.3 (**C**) and Lig.4 (**D**) with aromatase enzyme.

From Fig. 12 (A, B), it's clear that each molecule establishes strong interactions with the essential amino acids of the enzyme. The findings emphasize the efficacy of the active molecule when it exhibits low binding energy as well as a significant presence of electrostatic, steric, and hydrogen bond interactions with the receptor. Next, Lig. 3 and Lig. 4 were docked into the protein's active site, using the same 3D grid (Fig. 13 (C, D)).

By comparison with Mol. 34, the selected ligands (Lig. 3 and Lig. 4) exhibited a significant number of HBA and HBD interactions, as well as enhanced electrostatic interactions, particularly of the Pi-Sulfur type. Steric interactions were also found to play a role in the efficacy of cervical cancer drug-candidates. Furthermore, these ligands offered an additional advantage in the form of robust halogen bonds formed with essential amino acids, due to the electronegative properties of the Cl atom. Table 9 depicts the binding energies and interactions of Lig. 3 and Lig. 4, as well as Mol. 34 and Mol. 21 with aromatase.

**Table 9:** Docking Results for the best designed ligands as well as Mol. 34 and Mol. 21 as References.

Ligand	Binding Energy (Kcal/mol)	Hydrogen-binding interactions (HBD and HBA)		Electrostatic Interactions	Halogen Interactions	Steric//Hydrophobic interactions
		HBD//Pi	HBA//C	Pi-S//Pi- ion	Cl	Pi-Pi//Pi-Alkyl //Pi-Sigma
Lig. 3	−9.900	Asp 309; Leu 477; 2 of Leu 372**//**Cys 437; Thr 310	Met 374; Arg 115; Thr 310; Leu 372	Phe 134; 2 of Cys 437	Cys 437; Val 370; Phe 430; Met 364; Pro 429	Ser 478; Val 370; Ile 133; Ile 132; Ala 306; Phe 148; Ala 438; Phe 221; Trp 224; Leu 477; 3 of Val370
Lig. 4	−9.700	Ile 133 **//** Ala 438	Arg 115; 2of Arg 435; Arg 145; Trp 141; Gly 439	Met 374; 3 Cys 437; Trp 224 **//** Arg 115	Phe 430; Met 364; Val 370; Pro 429; Thr 310; Cys 437	Phe 134; Ile 132; Gly 436; Gly 431; Leu 477; Val 373; Leu 372; Ile 133; Va l370; Phe 430; Pro 429
Mol. 34	−9.100	Cys 437; 2 of Thr 310; Ala 306 **//** Ala 438	Arg 115 **//** Ser 478; Thr 310	Cys 437	Trp224; Leu 477; Val 369; Leu 372	Ile 133; Ile 132; Ala 307; Trp 224; Phe 221; Leu 477; Val 370
Mol. 21	−8.500	Thr 310	Cys 437	2 Cys 437	–	Val 373; Ala 306; Ile 133; Ile 132; Phe 148; Met 364; Phe 430; Pro 429; Val 370

From Table 9, the Mol. 34 has five typical hydrogen bond donors and acceptors and four halogen interactions with crucial amino acids. Cys 437 also has an electrostatic Pi-Sulfur interaction. Via seven pi-pi, Pi-alkyl and pi-sigma interactions, the heterocyclic and benzocyclic rings interact sterically and hydrophobically. Nevertheless, Mol. 21 docks in this active site through the following interactions: Two hydrogen bond interactions, two Pi-Sulfur interactions, and nine pi-pi, alkyl and pi-Sigma interactions. Comparing the values of binding energies and the number of these interactions might therefore explain the lower IC_50_ value of the most active molecules (Mol. 34, Lig. 3 and Lig. 4) in comparison with Mol. 21. Therefore, increasing the rate of Pi-Sulfur and hydrogen bond interactions, as well as the number of halogen and steric interactions in the modified (R) group, may have a significant impact on pIC_50_. On the basis of these observations, the previous findings obtained from CoMSIA remain well justified, which means that the docking results of the proposed new potent drug-candidates can also be reliably interpreted. In fact, it's clear that the designed molecules (Lig. 3 and Lig. 4) demonstrated significantly lower binding energies than Mol. 34 when subjected to docking in the same active site of aromatase (ID: 3EQM). These docking scores may also explain why Lig. 3 and Lig. 4, which were developed using a similar approach, have higher pIC_50_ values than Mol. 34. Thus, the more significant anti-cervical cancer activity demonstrated by these designed ligands emphasizes the importance of the number and kind of crucial interactions formed in the receptor's active site including the prevalence of Pi-Sulfur interactions, in particular with the S atom of the ligand and Cys 374 of the protein as well as halogen interactions via the Cl atom. Therefore, these molecular docking results are in agreement with the previous 2D/3D-QSAR results, as well as with the experimental findings that form the database of this investigation.

We notice that, although it is a powerful tool for predicting ligand-receptor interactions, molecular docking often requires detailed information on a single three-dimensional structure of the target protein, which limits the generalization of these interactions and their exploitation for screening a number of receptors responsible for inhibiting cervical cancer or other diseases. To successfully overcome these limitations and ensure the reliability of previous results, the implementation of ligand-based pharmacophore modelling may be an essential step. By integrating this approach with the molecular docking technique, the efficiency and precision of new drug development efforts may be enhanced by advancing the understanding of molecular interactions as well as the discovery of promising new treatments for cervical cancer.

### 3.6 Ligand-based pharmacophore models

The ligand-based approach to pharmacophore modeling offers many advantages, including its ability to exploit experimental data and its focus on the critical structures and chemical characteristics of active ligands. It is also highly advantageous in situations involving structurally diverse ligands, where a single compound may engage with several targets simultaneously.

**Fig. 14:**
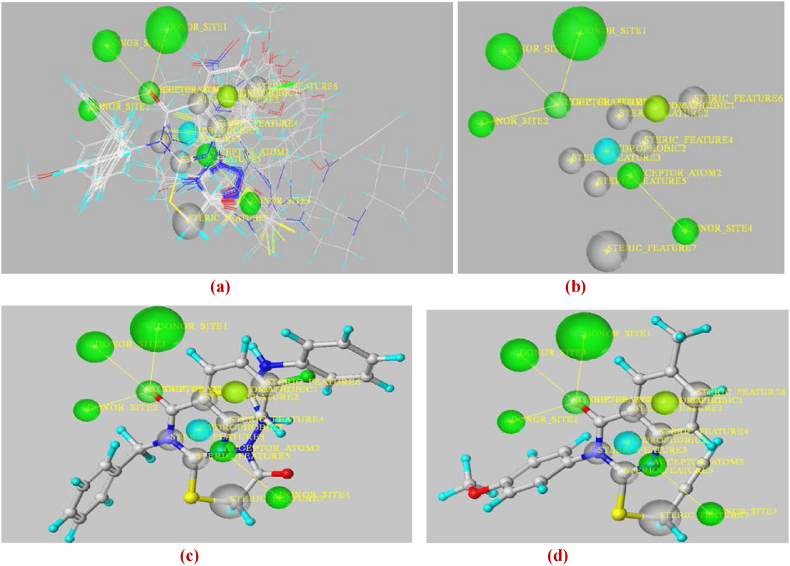
Pharmacophore of aligned molecules of training set (**a**), the validated model via test set (**b**), Mol. 34 features (**c**), and Mol. 21 features (**d**) (Green = hydrogen donor atom/acceptor site; cyan and yellow = hydrophobic and electrostatic interactions; Grey = steric interactions). (For interpretation of the references to colour in this figure legend, the reader is referred to the Web version of this article.)

The selection of relevant chemical features is one of the most crucial processes in pharmacophore development. Using ligand-based pharmacophore modeling, the chemical properties responsible for stopping cervical cancer may be identified. In this contribution study, the same training set (used in 2D/3D QSAR models) of 30 molecules based on their anti-cervical cancer activity information (pIC_50_) was chosen for the generation and evaluation of pharmacophore models using the Genetic algorithm implemented in SYBYL-X 2.0 software. All molecules were drawn in ChemOffice and then converted to Mol2 format using this program. Next, the Gasteiger-Huckel atomic partial charges were applied and the structures' energies were reduced using the Tripos Force Field (maximum iterations = 100000; convergence threshold = 10^−3^ kcal mol^−1^). The best pharmacophore model has been generated using the SYBYL-X 2.0 UNITY module and validated using the same previous test set (molecules:1-6-7-8-11-12-13-27-38). As a result, the essential features selected, which are necessary to be used against cervical cancer, were highlighted as shown in Fig. 14(a–d).

**Fig. 15:**
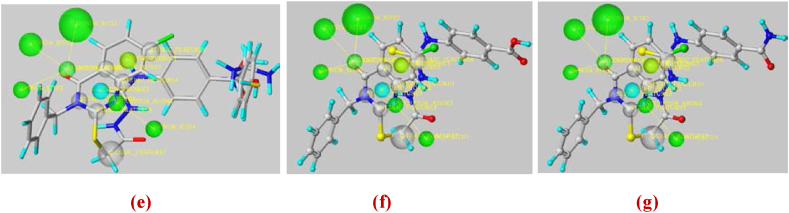
Pharmacophore generation of designed molecules (Lig. 1 - Lig. 6) (**e**), features of Lig. 4 (**f**) and features of Lig. 3 (**g**).

According to Fig. 14(a–d), the obtained model exhibits three mainly kinds of pharmacophore features that are hydrogen bond (HBA and HBD), steric, and hydrophobic. In addition, it's important to notice that HBA and HBD may contribute to the stability of the complex via polar interactions. This hypothesis was therefore adopted for the virtual screening using the most optimal pharmacophore model as a template for the flexible 3D alignment of the designed compounds. This alignment process was carried out using the UNITY module of SYBYL-X 2.0 in order to assess the potential of the compounds for elucidating structure-activity relationships for analogue molecules (test set as well as drug-candidates) which were not used in the development of the initial pharmacophore models. In order to anticipate their alignment with the pharmacophore features, two designed ligands (Lig. 3 and Lig. 4) were subjected to the same optimization protocol and individually aligned with the pharmacophore model using GALAHAD's flexible alignment mode (Fig. 15(e–g)).

The assessment findings indicate that hydrogen bonds (correlating with electrostatic effects), steric and hydrophobic interactions are the most effective for discovering novel active molecules against cervical cancer. These interactions are primarily important for the substrate/inhibitor complex's stability. As revealed by the findings of molecular docking, the majority of the most active derivatives (Mol. 34, Lig. 3 and Lig. 4) have the required groups on their main chain, which verifies the creation of hydrogen bonds (HBA/HBD) or electrostatic interactions. Therefore, the alignment of the potential drug-candidates with the features, also obtained via the CoMSIA model contour maps and via the visualization of docking interactions, suggests their potential to form strong and specific dockings with a critical protein target in cervical cancer (aromatase). This confirms the reliability of the pharmacophore model in validating the performance of molecular docking and the results obtained from the 2D/3D QSAR models.

As a result, the use of pharmacophore modelling offers a significant advantage by the simultaneous evaluation of multiple receptors, thereby significantly increasing the potential of promising drug-candidates for various diseases, following a comprehensive evaluation of their pharmacokinetic properties. Indeed, despite the significant improvements in the anti-cervical cancer efficacy of drug-candidates obtained through 2D/3D QSAR, docking and pharmacophore analyses, it is vital to emphasize the critical role of in silico ADMET prediction.

### 3.7 ADMET investigation

In silico evaluation of drug-candidates to determine their drug-likeness is a crucial step to be achieved before costly in vitro and in vivo experiments. Indeed, many potential therapeutic compounds are identified as unsuitable for clinical application during the development stages due to their unacceptable ADMET properties. Thus, using the online pkCSM tool, the pharmacokinetic properties of Lig. 3 and Lig. 4, in comparison with Mol. 34, were rigorously investigated (Table 10). Subsequently, priority will be given to compounds with properties similar to those of the most effective drugs [43].

**Table 10:** ADMET properties of Lig. 3, Lig. 4 and Mol. 34.

Pharmacokinetic parameters					Lig. 3	Lig. 4	Mol. 34
**Absorption**	Caco 2 permeability	**-**	**-**	P_app_ (10^−6^ cm/s)	0.521	0.547	0.934
	Intestinal absorption	**-**	**-**	% Absorbed	74.942	57.788	90.798
**Distribution**	VDss	**-**	**-**	Log (L/kg)	−0.606	−1.631	−0.461
	BBB	**-**	**-**	Log BB	−1.732	−1.924	−1.513
	CNS	**-**	**-**	Log PS	−2.661	−2.575	−2.198
**Metabolism**	CYP	Substrate	2D6	Yes/No	Yes	Yes	Yes
			3A4		Yes	Yes	Yes
		Inhibitor	1A2		No	No	Yes
			2C19		Yes	No	Yes
			2C9		Yes	Yes	Yes
			2D6		No	No	No
			3A4		Yes	No	Yes
**Excretion**	Total clearance	**-**	**-**	Log (mL min^−1^ kg^−1^)	−0.555	−0.35	−0.277
**Toxicity**	AMES toxicity	**-**	**-**	Yes/No	No	No	Yes

The Caco-2 permeability assay is a method used to assess the ability of substances to cross the barrier of human intestinal epithelial cells. As a result, the Caco-2 permeability levels for all the proposed compounds show notable values. Intestinal absorption results ranged from 0 % to 20 % (very poor absorption), from 20 % to 70 % (moderate absorption), from 70 % to 100 % (excellent absorption). Thus, the presence of the two selected ligands in the absorption norms of the human intestine suggests that they are absorbed. As numerous studies have shown that significant values of VDss (volume of distribution) are greater than 0.45, Lig. 3 and Lig. 4 have significant potential for achieving significant improvements in volume of distribution. Standard values for the permeability of substances via the blood-brain barrier (BBB) and the central nervous system (CNS) indicate that chemicals with LogBB values below −1 are poorly transported to the brain, while compounds with LogBB values over 0.3 have the potential to cross the BBB. In addition, while substances with a Log PS > −2 are expected to reach the CNS, those with a Log PS < −3 are considered to have difficulty crossing the CNS. In this case, none of the designed ligands (Lig. 3 and Lig. 4) can easily cross the barriers.

Among the CYP enzyme families (in particular CYP1A2, CYP2C9, CYP2C19, CYP2D6 and CYP3A4), which collectively regulate the biotransformation of over 90 % of drugs in the metabolic phase, CYP3A4 represents the most crucial enzyme. This suggests that all recently developed drug-candidates must have a mixed effect as CYP3A4 substrates or inhibitors. Accordingly, Lig. 3 and Lig. 4 were chosen as substrates or inhibitors of CYP3A4 whereas the reference molecule (Mol. 34) is neither a substrate nor an inhibitor of this metabolic enzyme. In addition, one measure of a drug's effectiveness is its clearance or rate of elimination from the body in relation to its blood levels. Thus, none of the new designed ligands in Table 10 presents a problem of drug retention. Finally, toxicity research is crucial throughout the early stages of a drug's development. In this investigation, the AMES test was used to assess the toxicity of each new drug-candidate, revealing that all the discovered ligands (Lig. 3 and Lig. 4) were non-toxic, while Mol. 34 showed toxicity after the cost of synthesis and experimental testing.

Based on the positive ADMET investigations and previous results (QSAR 2D/3D, Docking and pharmacophore), Lig. 3 and Lig. 4 have proved suitable for most of the molecular characteristics required in the search for the best drug candidates, suggesting their potential utility in the evaluation of cervical cancer inhibition both in vitro and in vivo as well as in the development of new anti-cancer drugs through structural modifications.

## 4 Conclusion

With the aim of reducing the cost of developing new drug-candidates against cervical cancer, this study integrated various computer-aided drug design (CADD) techniques, including ligand-based drug design (LBDD) and structure-based drug design (SBDD), in order to design new potent compounds from a series of 39 quinazoline and thioquinazolinone derivatives. Following a rigorous assessment process, including internal and external validation, as well as an evaluation of their domain of applicability, 2D/3D-QSAR predictive models were used to identify molecular descriptors that exert an influence on improvement of anti-cervical cancer activity. Descriptors characterized electronically (quantum descriptors), including dipolar moment (as well as molecular electrostatic potential), Mulliken charge and HOMO-1, were studied using density functional theory (DFT/B3LYP). Similarly, steric descriptors, characterized by the stretched bond (SB), were highlighted by in silico infrared spectroscopy. In addition, the number of hydrogen bond donor (NHD) and acceptor (NHA) was suitably checked by the CoMSIA model via HBD and HBA fields as well as the steric and electrostatic descriptors. These kinds of descriptors were further examined (as intermolecular interactions) by molecular docking simulations performed with the active site of an enzyme essential for inhibiting estrogen-associated cervical cancer (aromatase). Next, the pharmacophore model was used to corroborate the molecular docking results, reinforcing the reliability of the 2D/3D-QSAR models. In the light of these relevant results, six new molecules (named Lig. 1 to Lig. 6) were then designed with significant improvements in their efficacy against cervical cancer. In fact, these compounds showed strong interactions with crucial amino acids within the active site of the aromatase enzyme. Finally, ADMET in silico analyses also gave encouraging results, suggesting that only two drug-candidates (Lig. 3 and Lig. 4) have improved pharmacokinetic properties compared with the most potent molecule in the dataset (Mol. 34). These new ligands designed in silico could be rigorously tested in vitro and in vivo, offering multiple anti-cancer agents that could then be validated in experimental studies on the basis of their molecular structures.

## Funding statement

No external funding was obtained for this study.

## Data availability statement

Data will be made available on request.

## CRediT authorship contribution statement

**Said El Rhabori:** Writing – review & editing, Writing – original draft, Visualization, Validation, Supervision, Software, Resources, Project administration, Methodology, Investigation, Funding acquisition, Formal analysis, Data curation, Conceptualization. **Abdellah El Aissouq:** Visualization, Validation, Methodology, Investigation, Conceptualization. **Ossama :** Visualization, Validation, Investigation. **Souad :** Visualization, Validation, Investigation. **Samir Chtita:** Writing – review & editing, Visualization, Validation, Supervision, Methodology, Investigation. **Fouad Khalil:** Writing – review & editing, Visualization, Validation, Supervision, Methodology, Investigation, Formal analysis.

## Declaration of competing interest

The authors declare that they have no known competing financial interests or personal relationships that could have appeared to influence the work reported in this paper.

## References

[1] Bray F., Ferlay J., Soerjomataram I., Siegel R.L., Torre L.A., Jemal A. (2018). Global cancer statistics 2018: GLOBOCAN estimates of incidence and mortality worldwide for 36 cancers in 185 countries. CA A Cancer J. Clin..

[2] Siegel R.L., Miller K.D., Fuchs H.E., Jemal A. (2022). Cancer statistics, 2022, CA. A Cancer Journal for Clinicians.

[3] Šarenac T., Mikov M. (2019). Cervical cancer, different treatments and importance of bile acids as therapeutic agents in this disease. Front. Pharmacol..

[4] Zur Hausen H. (2002). Papillomaviruses and cancer: from basic studies to clinical application. Nat. Rev. Cancer.

[5] Chung S.H., Franceschi S., Lambert P.F. (2010). Estrogen and ERα: Culprits in cervical cancer?. Trends Endocrinol. Metabol..

[6] Zur Hausen H., Meinhof W., Scheiber W., Bornkamm G.W. (1974). Attempts to detect virus-specific DNA in human tumors. I. Nucleic acid hybridizations with complementary RNA of human wart virus. Int. J. Cancer.

[7] Di Domenico F., Foppoli C., Coccia R., Perluigi M. (2012). Antioxidants in cervical cancer: chemopreventive and chemotherapeutic effects of polyphenols. Biochim. Biophys. Acta (BBA) - Mol. Basis Dis..

[8] Hamid N.A., Brown C., Gaston K. (2009). The regulation of cell proliferation by the papillomavirus early proteins. Cell. Mol. Life Sci..

[9] Nair H.B., Luthra R., Kirma N., Liu Y.G., Flowers L., Evans D., Tekmal R.R. (2005). Induction of aromatase expression in cervical carcinomas: effects of endogenous estrogen on cervical cancer cell proliferation. Cancer Res..

[10] Mitrani-Rosenbaum S., Tsvieli R., Tur-Kaspa R. (1989). Oestrogen stimulates differential transcription of human papillomavirus type 16 in SiHa cervical carcinoma cells. J. Gen. Virol..

[11] Brake T., Lambert P.F. (2005). Estrogen contributes to the onset, persistence, and malignant progression of cervical cancer in a human papillomavirus-transgenic mouse model. Proc. Natl. Acad. Sci. U.S.A..

[12] Au W.W., Abdou-Salama S., Al-Hendy A. (2007). Inhibition of growth of cervical cancer cells using a dominant negative estrogen receptor gene. Gynecol. Oncol..

[13] Spurgeon M.E., Den Boon J.A., Horswill M., Barthakur S., Forouzan O., Rader J.S., Beebe D.J., Roopra A., Ahlquist P., Lambert P.F. (2017). Human papillomavirus oncogenes reprogram the cervical cancer microenvironment independently of and synergistically with estrogen. Proc. Natl. Acad. Sci. U.S.A..

[14] Marks M., Gravitt P.E., Gupta S.B., Liaw K.L., Kim E., Tadesse A., Phongnarisorn C., Wootipoom V., Yuenyao P., Vipupinyo C., Rugpao S., Sriplienchan S., Celentano D.D. (2011). The association of hormonal contraceptive use and HPV prevalence. Int. J. Cancer.

[15] Mitra S., Lami M.S., Ghosh A., Das R., Tallei T.E., Fatimawali, Islam F., Dhama K., Begum M.Y., Aldahish A., Chidambaram K., Bin Emran T. (2022). Hormonal therapy for gynecological cancers: how far has science progressed toward clinical applications?. Cancers.

[16] Rampogu S., Ravinder D., Pawar S.C., Lee K.W. (2018). Natural compound modulates the cervical cancer microenvironment—a pharmacophore guided molecular modelling approaches. J. Clin. Med..

[17] Hilborn E., Stål O., Jansson A., Hilborn E., Stål O., Jansson A. (2017). Estrogen and androgen-converting enzymes 17β-hydroxysteroid dehydrogenase and their involvement in cancer: with a special focus on 17β-hydroxysteroid dehydrogenase type 1, 2, and breast cancer. Oncotarget.

[18] Simpson E.R. (2003). Sources of estrogen and their importance. J. Steroid Biochem. Mol. Biol..

[19] Eckhardt S. (2002). Recent progress in the development of anticancer agents. Curr. Med. Chem. Anti Cancer Agents.

[20] Harlev E., Nevo E., Lansky E.P., Lansky S., Bishayee A. (2012). Anticancer attributes of desert plants: a review. Anti Cancer Drugs.

[21] El-Shafey H.W., Gomaa R.M., El-Messery S.M., Goda F.E. (2020). Quinazoline based HSP90 inhibitors: synthesis, modeling study and ADME calculations towards breast cancer targeting. Bioorg. Med. Chem. Lett.

[22] El-Shafey H.W., Gomaa R.M., El-Messery S.M., Goda F.E. (2020). Synthetic approaches, anticancer potential, HSP90 inhibition, multitarget evaluation, molecular modeling and apoptosis mechanistic study of thioquinazolinone skeleton: promising antibreast cancer agent. Bioorg. Chem..

[23] Hameed A., Al-Rashida M., Uroos M., Ali S.A., Arshia, Ishtiaq M., Khan K.M. (2018).

[24] Alagarsamy V., Chitra K., Saravanan G., Solomon V.R., Sulthana M.T., Narendhar B. (2018). An overview of quinazolines: pharmacological significance and recent developments. Eur. J. Med. Chem..

[25] Al-Rashood S.T., Hassan G.S., El-Messery S.M., Nagi M.N., Habib E.S.E., Al-Omary F.A.M., El-Subbagh H.I. (2014). Synthesis, biological evaluation and molecular modeling study of 2-(1,3,4-thiadiazolyl-thio and 4-methyl-thiazolyl-thio)-quinazolin-4-ones as a new class of DHFR inhibitors. Bioorg. Med. Chem. Lett.

[26] Sun D., Gao W., Hu H., Zhou S. (2022). Why 90% of clinical drug development fails and how to improve it?. Acta Pharm. Sin. B.

[27] Leelananda S.P., Lindert S. (2016). Computational methods in drug discovery. Beilstein J. Org. Chem..

[28] Ghosh D., Griswold J., Erman M., Pangborn W. (2009). Structural basis for androgen specificity and oestrogen synthesis in human aromatase. Nature.

[29] El Rhabori S., El Aissouq A., Chtita S., Khalil F. (2022). QSAR, molecular docking and ADMET studies of quinoline, isoquinoline and quinazoline derivatives against Plasmodium falciparum malaria. Struct. Chem..

[30] Daoui O., Elkhattabi S., Chtita S., Elkhalabi R., Zgou H., Benjelloun A.T. (2021). QSAR, molecular docking and ADMET properties in silico studies of novel 4,5,6,7-tetrahydrobenzo[D]-thiazol-2-Yl derivatives derived from dimedone as potent anti-tumor agents through inhibition of C-Met receptor tyrosine kinase. Heliyon.

[31] Spessard G.O. (1998). ACD labs/LogP dB 3.5 and ChemSketch 3.5. J. Chem. Inf. Comput. Sci..

[32] Hammoudan I., Matchi S., Bakhouch M., Belaidi S., Chtita S. (2021). QSAR modelling of peptidomimetic derivatives towards HKU4-CoV 3CLpro inhibitors against MERS-CoV. Chemistry.

[33] Rizk M.G., Emara A.A.A., Mahmoud N.H. (2021). Spectroscopic studies, DFT calculations, thermal analysis, anti-cancer evaluation of new metal complexes of 2-hydroxy-N-(4-phenylthiazol-2-yl)benzamide. J. Mol. Struct..

[34] Adekoya O.C., Adekoya G.J., Sadiku E.R., Hamam Y., Ray S.S. (2022). Application of DFT calculations in designing polymer-based drug delivery systems: an overview. Pharmaceutics.

[35] Hsu Y.L., Huang P.Y., Chen D.T. (2014). Sparse principal component analysis in cancer research. Transl. Cancer Res..

[36] Martin T.M., Harten P., Young D.M., Muratov E.N., Golbraikh A., Zhu H., Tropsha A. (2012). Does rational selection of training and test sets improve the outcome of QSAR modeling?. J. Chem. Inf. Model..

[37] Goodarzi M., Freitas M.P., Jensen R. (2009). Feature selection and linear/nonlinear regression methods for the accurate prediction of glycogen synthase kinase-3β inhibitory activities. J. Chem. Inf. Model..

[38] Khanfar M.A., Taha M.O. (2013). Elaborate ligand-based modeling coupled with multiple linear regression and k nearest neighbor QSAR analyses unveiled new nanomolar mTOR inhibitors. J. Chem. Inf. Model..

[39] Chtita S., Ghamali M., Ousaa A., Aouidate A., Belhassan A., Taourati A.I., Masand V.H., Bouachrine M., Lakhlifi T. (2019). QSAR study of anti-Human African Trypanosomiasis activity for 2-phenylimidazopyridines derivatives using DFT and Lipinski's descriptors. Heliyon.

[40] Alexander D.L.J., Tropsha A., Winkler D.A. (2015). Beware of R2: simple, unambiguous assessment of the prediction accuracy of QSAR and QSPR models. J. Chem. Inf. Model..

[41] Liu R., Glover K.P., Feasel M.G., Wallqvist A. (2018). General approach to estimate error bars for quantitative structure-activity relationship predictions of molecular activity. J. Chem. Inf. Model..

[42] Dahiru T. (2011). P-Value, a true test of statistical significance? a cautionary note. Ann. Ib. Postgrad. Med..

[43] El Rhabori S., El Aissouq A., Chtita S., Khalil F. (2022). Design of novel quinoline derivatives as antibreast cancer using 3D-QSAR, molecular docking and pharmacokinetic investigation. Anti Cancer Drugs.

[44] El Aissouq A., Chedadi O., Bouachrine M., Ouammou A., Khalil F. (2022).

[45] Ashraf N., Asari A., Yousaf N., Ahmad M., Ahmed M., Faisal A., Saleem M., Muddassar M. (2022). Combined 3D-QSAR, molecular docking and dynamics simulations studies to model and design TTK inhibitors. Front. Chem..

[46] Tabti K., El Mchichi L., Moukhliss Y., Singh G., Sbai A., maghat H., bouachrine M., Lakhlifi T. (2022). CoMFA Topomer, CoMFA, CoMSIA, HQSAR, docking molecular, dynamique study and ADMET study on phenyloxylpropyl isoxazole derivatives for coxsackie virus B3 virus inhibitors activity. Moroc. J. Chem..

[47] Goudzal A., El Aissouq A., El Hamdani H., Hadaji E.G., Ouammou A., Bouachrine M. (2022).

[48] Deng L., Ma L., Cheng K.K., Xu X., Raftery D., Dong J. (2021). Sparse PLS-based method for overlapping metabolite set enrichment analysis. J. Proteome Res..

[49] Cheng H., Garrick D.J., Fernando R.L. (2017). Efficient strategies for leave-one-out cross validation for genomic best linear unbiased prediction. J. Anim. Sci. Biotechnol..

[50] Chirico N., Gramatica P. (2011). Real external predictivity of QSAR models: how to evaluate It? Comparison of different validation criteria and proposal of using the concordance correlation coefficient. J. Chem. Inf. Model..

[51] Rücker C., Rücker G., Meringer M. (2007). Y-randomization and its variants in QSPR/QSAR. J. Chem. Inf. Model..

[52] H. Hadni, M. Bakhouch, M. Elhallaoui, 3D-QSAR, molecular docking, DFT and ADMET studies on quinazoline derivatives to explore novel DHFR inhibitors, 10.1080/07391102.2021.2004233. 34825630

[53] Roy K., Kar S., Ambure P. (2015). On a simple approach for determining applicability domain of QSAR models. Chemometr. Intell. Lab. Syst..

[54] Roy K. (2015).

[55] Onodera K., Satou K., Hirota H. (2007). Evaluations of molecular docking programs for virtual screening. J. Chem. Inf. Model..

[56] Muegge I., Heald S.L., Brittelli D. (2001). Simple selection criteria for drug-like chemical matter. J. Med. Chem..

[57] El Rhabori S., El Aissouq A., Chtita S., Khalil F. (2022). 3D-QSAR, molecular docking and ADMET studies of thioquinazolinone derivatives against breast cancer. J. Indian Chem. Soc..

[58] Nguyen N.T., Nguyen T.H., Pham T.N.H., Huy N.T., Van Bay M., Pham M.Q., Nam P.C., Vu V.V., Ngo S.T. (2020). Autodock vina adopts more accurate binding poses but Autodock 4 forms better binding affinity. J. Chem. Inf. Model..

[59] Pérez C., Ortiz A.R. (2001). Evaluation of docking functions for protein-ligand docking. J. Med. Chem..

[60] Qing X., Lee X.Y., De Raeymaeker J., Tame J.R., Zhang K.Y., De Maeyer M., Voet A.R. (2014). Pharmacophore modeling: advances, limitations, and current utility in drug discovery. J. Recept. Ligand Channel Res..

[61] Taha M.O., Dahabiyeh L.A., Bustanji Y., Zalloum H., Saleh S. (2008). Combining ligand-based pharmacophore modeling, quantitative structure-activity relationship analysis and in silico screening for the discovery of new potent hormone sensitive lipase inhibitors. J. Med. Chem..

[62] Fan H.T., Guo J.F., Zhang Y.X., Gu Y.X., Ning Z.Q., Qiao Y.J., Wang X. (2018). The rational search for PDE10A inhibitors from Sophora flavescens roots using pharmacophoreand docking-based virtual screening. Mol. Med. Rep..

[63] Chen Y., Tian Y., Gao Y., Wu F., Luo X., Ju X., Liu G. (2020). In silico design of novel HIV-1 NNRTIs based on combined modeling studies of dihydrofuro[3,4-d]pyrimidines. Front. Chem..

[64] Fei J., Zhou L., Liu T., Tang X.Y. (2013). Pharmacophore modeling, virtual screening, and molecular docking studies for discovery of novel Akt 2 inhibitors. Int. J. Med. Sci..

[65] Alamri M.A., Alamri M.A. (2019). Pharmacophore and docking-based sequential virtual screening for the identification of novel Sigma 1 receptor ligands. Bioinformation.

[66] Yang H., Sun L., Wang Z., Li W., Liu G., Tang Y. (2018). ADMETopt: a web server for ADMET optimization in drug design via scaffold hopping. J. Chem. Inf. Model..

[67] Darvas F., Dormán G., Papp A. (2000). Diversity measures for enhancing ADME admissibility of combinatorial libraries. J. Chem. Inf. Comput. Sci..

[68] Pires D.E.V., Blundell T.L., Ascher D.B. (2015). pkCSM: PredictingSmall-molecule pharmacokinetic andToxicity properties using graph-based signatures. J. Med. Chem..

[69] Golbraikh A., Tropsha A. (2002). Predictive QSAR modeling based on diversity sampling of experimental datasets for the training and test set selection. J. Comput. Aided Mol. Des..

[70] Akinwande M.O., Dikko H.G., Samson A. (2015). Variance inflation factor: as a condition for the inclusion of suppressor variable(s) in regression analysis. Open J. Stat..

[71] Politzer P., Daiker K.C., Donnelly R.A. (1976). Molecular electrostatic potentials: a new approach to the study of the metabolic and carcinogenic activities of hydrocarbons. Cancer Lett..

[72] Kushwaha P.S., Mishra P.C. (2003). Molecular electrostatic potential maps of the anti-cancer drugs daunomycin and adriamycin: an ab initio theoretical study. J. Mol. Struct.: THEOCHEM.

[73] Cruz J.C., Hernández-Esparza R., Vázquez-Mayagoitia Á., Vargas R., Garza J. (2019). Implementation of the molecular electrostatic potential over graphics processing units. J. Chem. Inf. Model..

[74] Anastassopoulou J., Kyriakidou M., Malesiou E., Rallis M., Theophanides T. (2019). Infrared and Raman spectroscopic studies of molecular disorders in skin cancer. In.

[75] Hu X., Margadant F.M., Yao M., Sheetz M.P. (2017). Molecular stretching modulates mechanosensing pathways. Protein Sci..

[76] Irwin J.J., Sterling T., Mysinger M.M., Bolstad E.S., Coleman R.G. (2012).

[77] Ertl P., Rohde B. (2012). The Molecule Cloud - compact visualization of large collections of molecules. J. Cheminf..

[78] Ertl P., Schuffenhauer A. (2009). Estimation of synthetic accessibility score of drug-like molecules based on molecular complexity and fragment contributions. J. Cheminf..

[79] Karami T.K., Hailu S., Feng S., Graham R., Gukasyan H.J. (2022). Eyes on lipinski's rule of five: a new “rule of thumb” for physicochemical design space of ophthalmic drugs. J. Ocul. Pharmacol. Therapeut..

