# Supplementary material for: Design of new molecules against cervical cancer using DFT, theoretical spectroscopy, 2D/3D-QSAR, molecular docking, pharmacophore and ADMET investigations
Source: Heliyon. 2024 Jan 24;10(3):e24551. doi: 10.1016/j.heliyon.2024.e24551 (PMC10839811; doi:10.1016/j.heliyon.2024.e24551)
Supplement: Multimedia component 1 [file mmc1.docx]

**Supplementary Materials**

**Title:**  Design of new molecules against cervical cancer using DFT, theoretical spectroscopy, 2D/3D-QSAR, molecular docking, pharmacophore and ADMET investigations

**Author list:** Said El Rhabori^a*^, Abdellah El Aissouq ^a^, [Ossama Daoui](https://pubmed.ncbi.nlm.nih.gov/?term=Daoui+O&cauthor_id=34296007)^b^, [Souad Elkhattabi](https://pubmed.ncbi.nlm.nih.gov/?term=Elkhattabi+S&cauthor_id=34296007)^b^, Samir Chtita^c^ and Fouad Khalil^a^

**Supplementary Figures (S_1_-S_3_)**

**X= O; CH_2_; N-CH_2_CH_3_; N-CH_2_-CH = CH_2_; N-CO-CH_3_; N-COO-C(CH_3_)_3_; N-C_6_H_11_; N-C_4_H_3_N_2_; CH_2_; N-CH_3_; N-CH_2_CH_3_; N-CH_2_-CH = CH_2_; N-CO-CH_3_; N-C_6_H_11_; N-C_6_H_5_; N-(2-OC_2_H_5_) C_6_H_4_; N-C_4_H_3_N_2_**

**1-8**

**9**

**10-18**

**Fig. S_1_.** Structures of the studied quinazoline derivatives

**23-31**

**19-22**

**32**

**37-39**

**34-36**

**33**

**R_1_= Cl; CH_3_  R_2_= H; OCH_3_  R_3_= NO_2_; COOH; COOCH_3_  n=0 ;1**

**R= H; OCH3; Cl; 3-OH-4-MeO-Ph; 3,4,5-triMeO-Ph; 2-furanyl**

**Fig. S_2_.** Structures of the studied thioquinazolinone derivatives

**Fig. S_3_.** Carbon's charge linked to sulfur atom (Q), charge of C_1_ (Q_1_), charge of C_2_ (Q_2_) and bond distance between C and S (Bond C-S).

**Supplementary Tables (Table S_1_-Table S_3_)**

**Table S_1_.** ACP Correlation Matrix of the used Descriptors

| **Variables** | **pIC_50_** | **NHA** | **NHD** | **Log P** | **NRB** | **D M** | **E_HOMO_** | **E_LUMO_** | **W** | **Q** | **Q1** | **Q2** | **Bond C-S** | **E_HOMO-1_** | **E_LUMO+1_** | **SB** | **E _Torsion_** |
| --- | --- | --- | --- | --- | --- | --- | --- | --- | --- | --- | --- | --- | --- | --- | --- | --- | --- |
| **pIC_50_** | 1 | 0.103 | 0.502 | -0.115 | 0.065 | -0.427 | 0.098 | 0.122 | -0.217 | 0.360 | -0.041 | 0.204 | 0.282 | -0.099 | 0.010 | -0.175 | -0.294 |
| **NHA** |  | 1 | 0.130 | 0.124 | 0.078 | 0.061 | -0.220 | -0.400 | 0.351 | 0.184 | -0.210 | 0.404 | -0.161 | -0.236 | -0.494 | -0.413 | -0.427 |
| **NHD** |  |  | 1 | 0.139 | 0.448 | -0.049 | 0.135 | -0.033 | -0.111 | -0.027 | -0.488 | 0.269 | 0.469 | 0.320 | -0.218 | -0.323 | -0.622 |
| **Log P** |  |  |  | 1 | 0.566 | 0.437 | 0.155 | -0.222 | 0.149 | -0.147 | -0.151 | 0.143 | -0.384 | 0.316 | -0.384 | -0.014 | -0.433 |
| **NRB** |  |  |  |  | 1 | 0.504 | 0.321 | 0.064 | -0.055 | -0.221 | -0.132 | -0.206 | 0.077 | 0.738 | -0.221 | 0.336 | -0.207 |
| **D M** |  |  |  |  |  | 1 | 0.023 | -0.082 | 0.174 | -0.205 | 0.128 | -0.345 | -0.340 | 0.515 | -0.046 | 0.280 | 0.080 |
| **E_HOMO_** |  |  |  |  |  |  | 1 | 0.574 | -0.635 | -0.091 | 0.052 | -0.162 | 0.049 | 0.401 | 0.278 | 0.303 | -0.035 |
| **E_LUMO_** |  |  |  |  |  |  |  | 1 | -0.891 | -0.078 | 0.454 | -0.285 | 0.164 | 0.187 | 0.762 | 0.365 | 0.492 |
| **W** |  |  |  |  |  |  |  |  | 1 | 0.086 | -0.316 | 0.134 | -0.214 | -0.159 | -0.523 | -0.227 | -0.220 |
| **Q** |  |  |  |  |  |  |  |  |  | 1 | -0.107 | 0.420 | -0.020 | -0.157 | 0.205 | -0.373 | -0.208 |
| **Q_1_** |  |  |  |  |  |  |  |  |  |  | 1 | -0.333 | -0.262 | 0.013 | 0.486 | 0.448 | 0.656 |
| **Q_2_** |  |  |  |  |  |  |  |  |  |  |  | 1 | 0.047 | -0.363 | -0.290 | -0.752 | -0.628 |
| **Bond C-S** |  |  |  |  |  |  |  |  |  |  |  |  | 1 | 0.035 | 0.089 | 0.046 | -0.108 |
| **E_HOMO-1_** |  |  |  |  |  |  |  |  |  |  |  |  |  | 1 | 0.103 | 0.450 | 0.001 |
| **E_LUMO+1_** |  |  |  |  |  |  |  |  |  |  |  |  |  |  | 1 | 0.278 | 0.559 |
| **SB** |  |  |  |  |  |  |  |  |  |  |  |  |  |  |  | 1 | 0.656 |
| **E _Torsion_** |  |  |  |  |  |  |  |  |  |  |  |  |  |  |  |  | **1** |

**Table S_2_.** Statistical parameters of created model via random division of dataset

| **MLR models** | **R^2^** | **R^2^_adj_** | **MSE** | **F** | **VIF** | **R^2^_test_** | **R^2^_cv_** | **R^2^_yrand_** | **Q^2^_yrand_** | **cR_r_^2^** | **TEST SET** |
| --- | --- | --- | --- | --- | --- | --- | --- | --- | --- | --- | --- |
| **Model 1:**  **PIC50 =** 0.306+0.309***NHD+**0.088***NRB-**0.128***D M+**2.335***Q+**1.622***Q1-**2.316***Q2-**15.957***E_HOMO-1_** | 0.738 | 0.654 | 0.048 | 8.834 | ≤5 | 0.736 | 0.538 | 0.239 | -0.447 | 0.614 | 1-6-7-9-19-25-29-35-39 |
| **Model 2:**  **PIC50 =** 0.770**+**0.349***NHD+**0.074***NRB-**0.104***D M+**2.318***Q+1**.325***Q1-**1.781***Q2-**13.951***E_HOMO-1_** | 0.728 | 0.641 | 0.048 | 8.402 | ≤5 | 0.748 | 0.509 | 0.249 | -0.462 | 0.597 | 10-11-16-20-28-36-7-29-35 |
| **Model 3:**  **PIC50 =** 2.471**+**0.290***NHD+**0.075***NRB-**0.104***D M+**1.334***Q+**1.510***Q1-**8.252***E_HOMO-1_** | 0.716 | 0.642 | 0.041 | 9.662 | ≤5 | 0.685 | 0.503 | 0.214 | -0.383 | 0.608 | 3-8-12-21-23-25-35-36-38 |
| **Model 4:**  **PIC50** = 0.481+0.063***NHA**+0.334***NHD**-0.092***D M**+1.510***Q** +1.019***Q1**-15.868***E_HOMO-1_**+0.922***SB** | 0.779 | 0.709 | 0.037 | 11.081 | ≤5 | 0.937 | 0.586 | 0.238 | -0.432 | 0.658 | 1-6-7-8-11-12-13-27-38 |
| **Model 5:**  **PIC50=** 4.461**+**0.077***NHA+**0.241***NHD-**0.134***D M+**1.952***Q+**1.206***Q1-**2.652***Q2** | 0.742 | 0.675 | 0.042 | 11.044 | ≤5 | 0.896 | 0.584 | 0.205 | -0.369 | 0.638 | 1-6-7-8-11-12-13-27-38 |
| **Model 6:**  **PIC50 =** 0.730**+**0.076***NHA+**0.332***NHD-**0.110***D M+**1.929***Q+**1.078***Q1-**1.789***Q2-**14.436***E_HOMO-1_+**0.674***SB** | 0.820 | 0.751 | 0.032 | 11.916 | ≤5 | 0.742 | 0.539 | 0.276 | -0.516 | 0.644 | 1-6-7-8-11-12-13-27-38 |

**Table S_3_.** Selected descriptors and the predicted values of anti-cervical cancer activities via generated models (*: test set)

| **N°** | **PIC50** | **NHA** | **NHD** | **D M** | **Q** | **Q_1_** | **E_HOMO-1_** | **SB** | **E** | **HBD** | **HBA** | **S (Steric)** | **pIC_50_**  **(MLR)** | **pIC_50_ (CoMSIA)** |  |
| --- | --- | --- | --- | --- | --- | --- | --- | --- | --- | --- | --- | --- | --- | --- | --- |
| **1*** | 4.739 | 4 | 0 | 2.317 | 0.178 | -0.120 | -0.245 | 0.405 | 1.154 | 0.000 | 4.408 | 6.550 | 4.833 | 4.736 | |
| **2** | 4.648 | 3 | 0 | 3.554 | 0.145 | -0.119 | -0.249 | 0.295 | 0.903 | 0.000 | 3.292 | 6.735 | 4.662 | 4.726 | |
| **3** | 4.427 | 4 | 0 | 3.150 | 0.008 | -0.121 | -0.238 | 0.559 | 0.951 | 0.000 | 3.296 | 7.247 | 4.615 | 4.700 | |
| **4** | 4.874 | 4 | 0 | 2.985 | 0.178 | -0.120 | -0.237 | 0.513 | 0.961 | 0.000 | 3.296 | 7.386 | 4.844 | 4.725 | |
| **5** | 4.920 | 4 | 0 | 1.194 | 0.008 | -0.121 | -0.243 | 0.425 | 1.133 | 0.000 | 3.914 | 6.898 | 4.756 | 4.949 | |
| **6*** | 4.462 | 4 | 0 | 1.787 | 0.008 | -0.121 | -0.247 | 0.667 | 1.216 | 0.000 | 4.336 | 7.483 | 4.684 | 4.525 | |
| **7*** | 4.271 | 4 | 0 | 3.603 | -0.009 | -0.118 | -0.240 | 0.661 | 1.125 | 0.000 | 3.713 | 7.713 | 4.291 | 4.270 | |
| **8*** | 4.114 | 6 | 0 | 3.606 | -0.008 | -0.117 | -0.245 | 0.493 | 1.223 | 0.000 | 4.284 | 7.092 | 4.129 | 4.213 | |
| **9** | 4.778 | 2 | 0 | 1.540 | -0.007 | -0.117 | -0.256 | 0.162 | 0.607 | 0.000 | 1.731 | 5.519 | 4.541 | 4.702 | |
| **10** | 4.299 | 2 | 0 | 5.459 | -0.009 | -0.118 | -0.237 | 0.318 | 0.783 | 0.000 | 1.912 | 6.790 | 4.018 | 4.345 | |
| **11*** | 4.477 | 3 | 0 | 5.233 | 0.158 | -0.119 | -0.220 | 0.485 | 0.948 | 0.000 | 2.171 | 6.938 | 4.438 | 4.487 | |
| **12*** | 4.099 | 3 | 0 | 5.304 | -0.009 | -0.117 | -0.230 | 0.552 | 0.977 | 0.000 | 2.174 | 7.134 | 4.104 | 4.019 | |
| **13*** | 4.543 | 3 | 0 | 4.945 | 0.158 | -0.119 | -0.220 | 0.522 | 0.966 | 0.000 | 2.166 | 7.176 | 4.596 | 4.488 | |
| **14** | 4.261 | 3 | 0 | 4.328 | -0.009 | -0.117 | -0.239 | 0.447 | 1.034 | 0.000 | 2.820 | 7.034 | 4.336 | 4.202 | |
| **15** | 4.190 | 3 | 0 | 5.586 | -0.009 | -0.118 | -0.229 | 0.839 | 0.851 | 0.000 | 1.971 | 7.829 | 4.436 | 4.253 | |
| **16** | 4.583 | 3 | 0 | 4.170 | -0.008 | -0.117 | -0.229 | 0.701 | 0.901 | 0.000 | 1.914 | 7.324 | 4.431 | 4.548 | |
| **17** | 4.687 | 4 | 0 | 6.050 | -0.008 | -0.118 | -0.223 | 0.922 | 0.922 | 0.000 | 1.905 | 7.762 | 4.434 | 4.680 | |
| **18** | 4.128 | 5 | 0 | 5.597 | -0.009 | -0.117 | -0.230 | 0.527 | 0.950 | 0.000 | 2.747 | 7.204 | 4.274 | 4.154 | |
| **19** | 4.143 | 3 | 0 | 3.758 | 0.169 | -0.290 | -0.244 | 0.151 | 0.717 | 0.000 | 1.733 | 5.438 | 4.300 | 4.121 | |
| **20** | 4.309 | 2 | 0 | 2.274 | 0.066 | -0.304 | -0.249 | 0.153 | 0.678 | 0.000 | 1.734 | 5.403 | 4.277 | 4.282 | |
| **21** | 4.038 | 3 | 0 | 1.946 | 0.006 | -0.106 | -0.239 | 0.140 | 0.746 | 0.000 | 1.733 | 5.636 | 4.314 | 4.122 | |
| **22** | 4.153 | 2 | 0 | 2.344 | 0.067 | -0.105 | -0.240 | 0.138 | 0.716 | 0.000 | 1.735 | 5.611 | 4.321 | 4.284 | |
| **23** | 4.069 | 7 | 0 | 4.801 | 0.043 | -0.306 | -0.251 | 0.087 | 1.305 | 0.000 | 3.332 | 6.872 | 4.294 | 4.113 | |
| **24** | 4.840 | 7 | 1 | 1.615 | 0.000 | -0.303 | -0.251 | 0.051 | 1.241 | 0.695 | 3.563 | 6.958 | 4.826 | 4.794 | |
| **25** | 4.374 | 6 | 0 | 5.059 | 0.175 | -0.294 | -0.236 | 0.086 | 1.307 | 0.000 | 3.346 | 6.812 | 4.188 | 4.291 | |
| **26** | 4.971 | 6 | 1 | 2.179 | 0.105 | -0.300 | -0.243 | 0.052 | 1.219 | 0.682 | 3.550 | 6.798 | 4.751 | 5.047 | |
| **27*** | 4.175 | 6 | 0 | 1.678 | 0.105 | -0.300 | -0.244 | 0.154 | 1.486 | 0.000 | 3.833 | 6.897 | 4.173 | 4.180 | |
| **28** | 5.003 | 7 | 1 | 2.239 | 0.194 | -0.111 | -0.238 | 0.039 | 1.263 | 0.692 | 3.561 | 7.057 | 5.040 | 4.922 | |
| **29** | 4.242 | 6 | 0 | 4.921 | 0.018 | -0.101 | -0.248 | 0.024 | 1.315 | 0.000 | 3.571 | 6.720 | 4.284 | 4.287 | |
| **30** | 5.122 | 6 | 1 | 3.004 | 0.182 | -0.109 | -0.259 | -0.015 | 1.214 | 0.681 | 3.711 | 6.794 | 5.179 | 5.141 | |
| **31** | 5.040 | 6 | 0 | 2.161 | 0.182 | -0.109 | -0.256 | 0.086 | 1.270 | 0.000 | 3.451 | 6.911 | 4.957 | 5.018 | |
| **32** | 4.336 | 3 | 0 | 3.633 | 0.064 | -0.303 | -0.249 | 0.390 | 0.875 | 0.000 | 2.766 | 6.109 | 4.432 | 4.322 | |
| **33** | 5.071 | 4 | 2 | 3.101 | 0.055 | -0.303 | -0.245 | 0.190 | 0.950 | 0.963 | 3.155 | 5.850 | 4.955 | 5.109 | |
| **34** | 5.170 | 3 | 3 | 2.110 | 0.048 | -0.303 | -0.221 | 0.230 | 1.057 | 1.676 | 3.191 | 6.889 | 4.963 | 5.120 | |
| **35** | 4.715 | 4 | 3 | 3.217 | 0.048 | -0.303 | -0.223 | 0.282 | 1.141 | 1.714 | 3.063 | 7.366 | 4.993 | 4.783 | |
| **36** | 4.855 | 3 | 3 | 3.146 | 0.048 | -0.303 | -0.228 | 0.239 | 1.087 | 1.709 | 3.043 | 7.170 | 4.989 | 4.804 | |
| **37** | 4.550 | 6 | 2 | 7.621 | -0.009 | -0.118 | -0.230 | 0.151 | 1.155 | 1.206 | 2.479 | 7.090 | 4.475 | 4.520 | |
| **38*** | 4.138 | 7 | 1 | 5.233 | 0.056 | -0.303 | -0.231 | 0.149 | 1.411 | 1.120 | 2.419 | 7.889 | 4.1481 | 4.131 | |
| **39** | 4.252 | 5 | 1 | 5.304 | 0.056 | -0.303 | -0.232 | 0.073 | 1.058 | 0.981 | 2.264 | 6.855 | 4.163 | 4.288 | |
